# ΔNp63α-induced DUSP4/GSK3β/SNAI1 pathway in epithelial cells drives endometrial fibrosis

**DOI:** 10.1038/s41419-020-2666-y

**Published:** 2020-06-11

**Authors:** Guangfeng Zhao, Ruotian Li, Yun Cao, Minmin Song, Peipei Jiang, Qianwen Wu, Zhenhua Zhou, Hui Zhu, Huiyan Wang, Chenyan Dai, Dan Liu, Simin Yao, Haining Lv, Limin Wang, Jianwu Dai, Yan Zhou, Yali Hu

**Affiliations:** 1 Department of Obstetrics and Gynecology, The Affiliated Drum Tower Hospital of Nanjing University Medical School, 321 Zhongshan Rd., Nanjing, 210008 China; 2 Department of Laboratory Medicine, Jiangsu Key Laboratory for Molecular Medicine, Nanjing University Medical School, 321 Zhongshan Rd., Nanjing, 210008 China; 3 Department of Obstetrics and Gynecology, Nanjing Drum Tower Hospital, Peking Union Medical College, Chinese Academy of Medical Science, Graduate School of Peking Union Medical College, Nanjing, 210008 China; 4 Department of Obstetrics and Gynecology, Drum Tower Clinical Medical College, Nanjing Medical University, Nanjing, 210000 China; 5 Institute of Genetics and Developmental Biology, Chinese Academy of Sciences, 3 Nanyitiao, Zhongguancun, Beijing, 100190 China; 6 Department of Obstetrics, Gynecology and Reproductive Sciences, Center for Reproductive Sciences, Eli and Edythe Broad Center of Regeneration Medicine and Stem Cell Research, University of California San Francisco, San Francisco, CA 94131 USA

**Keywords:** Molecular biology, Reproductive disorders

## Abstract

Epithelial homeostasis plays an essential role in maintaining endometrial function. But the epithelial role in endometrial fibrosis has been less studied. Previously, we showed that ectopic expression of ΔNp63α is associated with fibrosis process and epithelial dysfunction in endometria of patients with intrauterine adhesions (IUAs). Since ΔNp63α is profoundly involved in maintaining the epithelial homeostasis, we hereby focused on its roles in regulating the function and phenotype of endometrial epithelial cells (EECs) in context of endometrial fibrosis. We identified a typical type 2 epithelial-to-mesenchymal transition (EMT) in EECs from IUA patients and this process was induced by the forced expression of ΔNp63α in EECs. In transcriptomic analysis, we found that diverse signaling pathways regulated by ΔNp63α were involved in pro-EMT. We demonstrated that the DUSP4/GSK-3β/SNAI1 pathway was critical in transducing the pro-EMT signals initiated by ΔNp63α, while bFGF reversed ΔNp63α-induced EMT and endometrial fibrosis both in vitro and in vivo by blocking DUSP4/GSK3β/SNAI1 pathway. Taken together, our findings are important to understand the molecular mechanisms of endometrial fibrosis and to provide potential therapeutic targets.

## Introduction

Epithelial homeostasis is essential for tissue function^1,2^. Loss of the epithelial homeostasis leads to tissue remodeling and dysfunction^3^. In spite of cyclical endometrial shedding and rehealing in every menstrual cycle, the epithelial homeostasis in endometrium is well-developed and maintained. The mechanism to maintain the relatively stable epithelial phenotype in endometrial epithelial cells (EECs) remains largely unknown^4^. Since epithelium is directly exposed to exogenous injuries, such as intrauterine procedures, it is likely that these injuries disrupt the epithelial homeostasis and result in local tissue remodeling and dysfunction^5^. Endometrial fibrosis is the commonest pathology following intrauterine procedures and its mechanisms remain elusive^6^. Especially, the contributions of epithelial dysfunction and the molecular processes involved in the epithelial dysfunction have not well been characterized. The usual phenotype of disrupted epithelial homeostasis is epithelial–mesenchymal-transition (EMT), in which the epithelial cells have the changed phenotype and lose the polarity, while the cells gain the mesenchymal phenotype and motility^7^. Given that EMT is closely involved in the fibrosis of other organs such as kidneys and lungs, we hypothesized that EMT might be involved in the development of endometrial fibrosis.

Previously, we revealed that a ΔNp63α + lineage emerges in the endometria of patients with IUAs^8^. ΔNp63α is the main form of p63 in epithelial cells^9^. It is known that during uterine development, p63 is expressed in the epithelial cells of the paramesonephric ducts but is no longer expressed in EECs after the fusion of bilateral paramesonephric ducts^10^. The expansion of the p63 lineage in pathological conditions is associated with pulmonary fibrotic lesions^11,12^, suggesting that p63 functions in maintaining the homeostasis of epithelial tissues and is likely associated with fibrotic lesions. However, it is unclear how the ectopic expression of p63 in endometrium affects the epithelial homeostasis. As we found that the administration of autologous bone marrow stem cells to the uterine cavity in IUA patients reduced the ΔNp63α + lineage, restored the endometria response to sex hormones, alleviated endometrial fibrosis, attained successful pregnancy, and finally delivered term babies^8^, we hypothesized that ΔNp63α may promote endometrial fibrosis by driving EMT, and if it does, the therapy with antagonizing ΔNp63α could restore functional endometria by reversing EMT.

To test our hypotheses, we conducted various experiments in vitro and in vivo, and in IUA patients. We demarcated the roles of type 2 EMT in fibrotic endometria, and identified a novel signal pathway DUSP4/GSK3β/Snai1. Moreover, we demonstrated that tissue regeneration factor, basic fibroblast growth factor (bFGF), could antagonize the effects of ΔNp63α and reverse EMT and endometrial fibrosis. The present study advances our understanding of cellular and molecular mechanisms of endometrial fibrosis and provides insights into novel therapeutic targets.

## Materials and methods

### Patients and clinical information

Patients with IUA who underwent hysteroscopy adhesiolysis at the Affiliated Drum Tower Hospital of Nanjing University from January 2014 to December 2016. Written informed consent was obtained from each participant. This study was approved by the Committee on Human Research of the Nanjing Drum Tower Hospital (No. 2012022). The diagnosis of IUA was based on criteria recommended by the American Fertility Society^13^. Totally, 69 patients with severe IUA were initially included. Of them, 16 had only detectable mRNA of ΔNp63α by quantitative polymerase chain reaction (qPCR), 2 were positive ΔNp63α protein only in IHC, 30 were positive both in qPCR and IHC, and remaining 21 had undetectable mRNA and protein in qPCR and IHC. Those 30 patients with detectable mRNA and protein were selected in the further experiments. The patients had normal ovary function. The control group was comprised of 30 patients with a normal endometrium and ovaries, as assessed by hysteroscopy and ultrasonography during infertility screening. The clinical information of all participants is summarized in Supplemental Table 1.

### RNA-seq read processing, assembly, and transcriptome sequence analysis

For the RNA-seq of EECs, total RNA was prepared using an RNeasy Plus Micro Kit (Qiagen, Dusseldorf, Germany). The RNA purity was assessed using a Nano Drop spectrophotometer, the RNA concentration was assessed using a Qubit 3.0 fluorometer and the RNA integrity was assessed using an Agilent 2100 Bioanalyzer. The sequencing library was prepared following the instruction manual of a VAHTS total RNA-seq (H/M/R) Library Prep Kit for Illumina^®^ (Vazyme, China). To isolate the appropriate cDNA fragment size for sequencing, the library fragments were selected with VAHTSTM DNA Clean Beads. The enzyme UDG was used to digest the second strand of cDNA. PCR amplification was performed, and the products were purified. After cluster generation, the libraries were sequenced on an Illumina Hiseq X10 platform (Illumina, California, USA), and 150-bp paired-end reads were generated. Raw reads in the FASTQ format were first processed using in-house Perl scripts. Then, the processed reads were mapped to the reference genome. The reference genome and gene model annotation files were downloaded directly from the genome website. The reference genome index was built using hisat2-build^14^, and paired-end clean reads were aligned to the reference genome using Hisat2 (v2.0.5)^15^.

### mRNA differential expression analysis

Cuffdiff (v1.3.0)^16^ was used to calculate the fragments per kilobase of exon per million reads mapped (FPKMs) for coding genes in each sample. Gene FPKMs were computed by summing the FPKMs of the transcripts in each gene group, and FPKMs were calculated based on the length of the fragments and the read counts mapped to each fragment. Cuffdiff (v2.2.1)^16^ provides statistical methods for determining differential expression in digital transcript or gene expression datasets using a model based on a negative binomial distribution. Transcripts or genes with corrected p values less than 0.05 and an absolute value of log2 (fold change) >1 were defined as being significantly differentially expressed gene (DEG). Heatmaps were generated using pheatmap in R principal component analysis. Gene ontology (GO) enrichment analysis of DEGs was performed with a Perl module (GO::TermFinder). GO terms with corrected *p* values less than 0.05 were significantly enriched among the DEGs.

### IUA-like mouse model

Animal experiments were approved by the Institutional Animal Care and Use Committee at the Nanjing Drum Tower Hospital, Nanjing University Medical School. Eight-week-old BALB/c female virgin mice, weighing 18–20 g, were purchased from the Experimental Animal Center of Nanjing Medical University (Nanjing, China). Mouse model of endometrial fibrosis was established by dual (mechanical and inflammation) methods using uterine curettage and lipopolysaccharide (LPS) injection as previously described^17^. Briefly, the mouse model was created at estrum, which corresponds to the late reproductive phase in humans, based on vaginal smears^18,19^. All the mice were anesthetized with 4% chloral hydrate (10 mg/kg) through intraperitoneal injections. The uterus was exposed and the horn was damaged with a rough surface needle that was inserted all the way through the lumen and scratched up and down for 2 min until the uterine wall became rough. A single dose of 20 μl LPS (10 mg/ml, derived from *Escherichia coli* 0111: B4; Sigma, St. Louis, MO, USA) was administered via intrauterine injection to cause endometrial injury, and the ends were clamped with tweezers for 5 min. A vertical incision was made in the abdominal wall of control mice. Mouse models were randomly assigned at a 1:1:1 ration into three groups: (a) the sham group, (b) the PBS group, and (c) the bFGF treatment group. The treatment groups received 2 intrauterine injections with 20 μl PBS or bFGF (100 mg/kg) 7 days apart, and the ends were clamped with tweezers for 5 min. After the surgery, mice were intramuscularly injected (thigh) with penicillin (20,000 U/day) for 3 days. Mice were sacrificed at the fifth estrous periods (approximately 28 days) after injury and all the uteruses were collected at estrum.

Some specimens from each group were fixed in formaldehyde and embedded in paraffin. Five-micron sections were cut and stained with hematoxylin and eosin (H&E) as well as Masson stain to evaluate the histological evidence of fibrosis in a blinded manner. Other specimens were used for quantitative real time PCR and western blotting. The antibodies used in this study are listed in Supplemental Table 2.

### Statistics

Statistical analyses were performed using GraphPad Prism software (version 5.01, San Diego, CA). The data are presented as the mean ± standard deviation for the number of independent experiments indicated in each figure legend. One-way ANOVA followed by a Student–Newman–Keuls multiple comparisons test were used to compare three or more experimental groups. A Student’s *t* test was used for comparisons of two experimental groups when the data were normally distributed. When the data were not normally distributed, a nonparametric test was used. Statistical significance was defined as *p* < 0.05.

More details of Materials and methods are provided in Supplementary materials.

## Results

### ΔNp63α lineage expansion is associated with EEC–EMT in the endometrium of patients with IUA

Endometrial fibrosis in IUA patients is characterized with scaring under hysteroscopic observation, however, it is not clear whether luminal epithelium slough from the mesenchymal layer or whether these cells lose their epithelial properties. To clarify this issue, we performed the various experiments in the endometrial sections from 30 patients who showed significant elevation of mRNA and protein of ΔNp63α in endometrial biopsy.

**Fig. 1:**
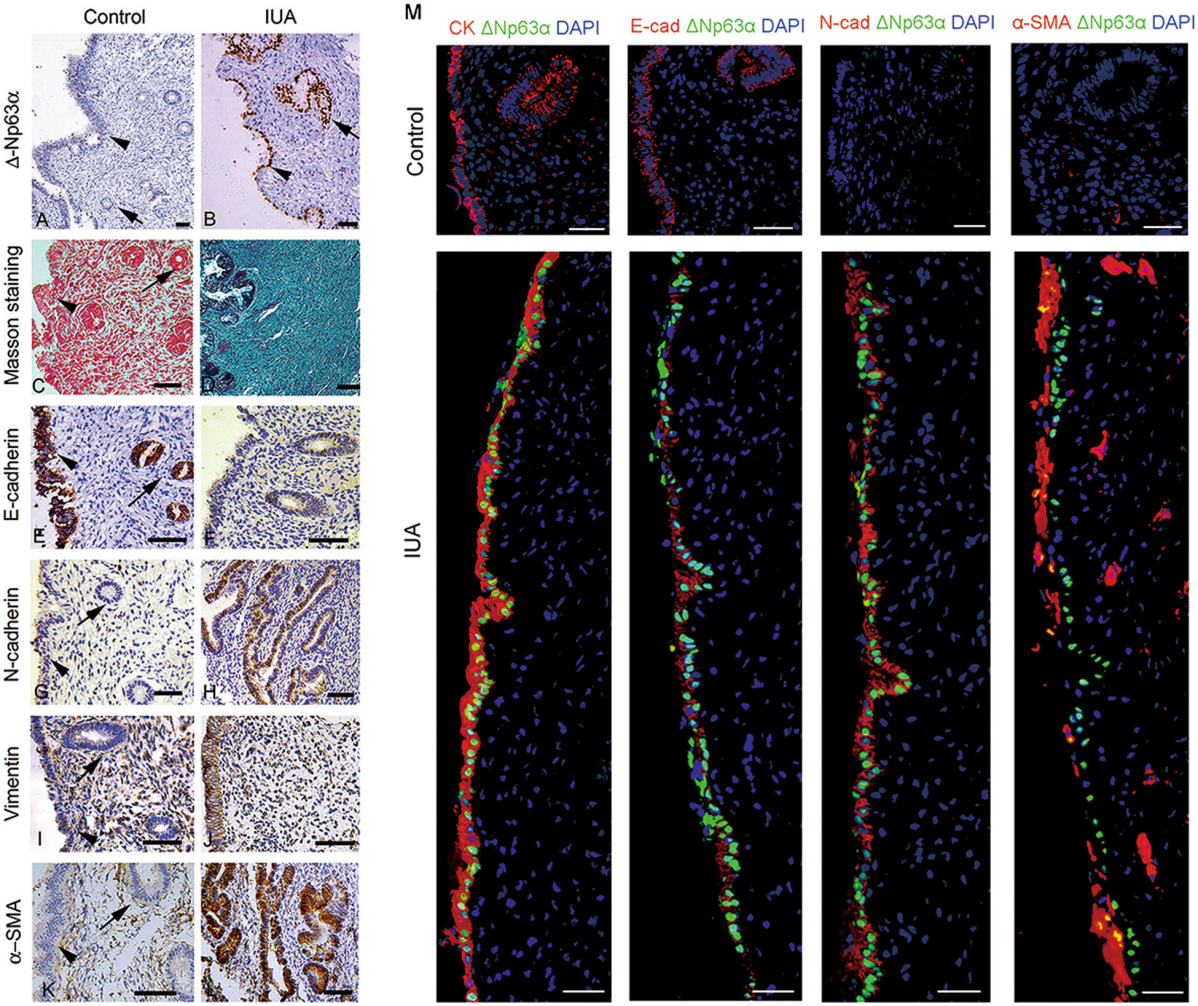
EEC–EMT is associated with ΔNp63α upregulation in endometrial biopsies from IUA patients. **a**, **b** Representative images of ΔNp63α immunostaining in endometrial biopsies of normal (*n* = 30) and fibrotic endometria (*n* = 30). **c**, **d** Representative images of Masson staining in normal (*n* = 30) and fibrotic endometria (*n* = 30). **e**, **f** Representative images of E-cadherin immunostaining in endometrial biopsies of normal (*n* = 30) and fibrotic endometria (*n* = 30). **g**, **h** Representative images of N-cadherin immunostaining in endometrial biopsies of normal (*n* = 30) and fibrotic endometria (*n* = 30). **i**, **j** Representative images of Vimentin immunostaining in endometrial biopsies of normal (*n* = 30) and fibrotic endometria (*n* = 30). **k**, **l** Representative images of α-SMA immunostaining in endometrial biopsies of normal (*n* = 30) and fibrotic endometria (*n* = 30). Scale bars, 50 μm. Arrowhead: luminal epithelial cells; arrow: glandular epithelial cells. **m** Representative images of colocation of ΔNp63α with cytokeratin (CK), E-cadherin, N-cadherin, or α-SMA in endometrial biopsies of normal (*n* = 5) and fibrotic endometria (*n* = 5). Scale bars, 25 μm.

We compared the distribution of the ΔNp63α-positive lineage in the luminal and glandular epithelia of endometria by immunohistochemistry. ΔNp63α + lineage presented focal distribution. As depicted in Fig. 1a, b and Supplementary Fig. 1A, the ΔNp63α + lineage could expand along the luminal epithelium of the endometrium, or in the glandular epithelium. Overall, 40 ΔNp63α positive foci were identified on the endometrial sections prepared from the 30 IUA patients. Of 40 ΔNp63α positive foci, 25 were located in the luminal epithelial regions, 4 were located in the glandular epithelial regions, and 11 were located in both the luminal and glandular epithelial regions. To further confirm whether ΔNp63α is expressed in epithelial cells, immunofluorescence co-localization of ΔNp63α and CK was performed and the results showed that ΔNp63α and CK were located in the same cells by confocal laser microscopy (Supplementary Fig. 2).

Masson’s trichrome staining showed that collagen staining was detected not only in endometrial stroma, but also in luminal EECs in IUAs, suggesting that EECs lost their epithelial properties and gained mesenchymal features (Fig. 1c, d, Supplementary Fig. 1B).

To examine whether the endometrial epithelium undergoes EMT in IUAs, we analyzed the expression of CK, E-cadherin, N-cadherin, α-smooth muscle actin (α-SMA), and vimentin in endometrial biopsies. As shown in Fig. 1e–l, while EECs remained to express CK (Supplementary Fig. 1C), E-cadherin was globally downregulated on luminal and glandular EECs (Fig. 1e, f), but N-cadherin was intensively upregulated on both epithelial and stromal cells (Fig. 1g, h). Vimentin, a hallmark of stromal cells, was also upregulated in epithelial cells (Fig. 1i, j). Notably, α-SMA was upregulated in both luminal and glandular epithelial cells (Fig. 1k, l). To verify the co-localization of ΔNp63α and CK, E-cadherin, N-cadherin, and a-SMA, we performed immunofluorescence staining and found that they were co-located in EEC with confocal microscopy (Fig. 1m). These phenotype changes were consistent with type 2 EMT.

### ΔNp63α enriches the transcription of genes that promote EMT in EECs

**Fig. 2:**
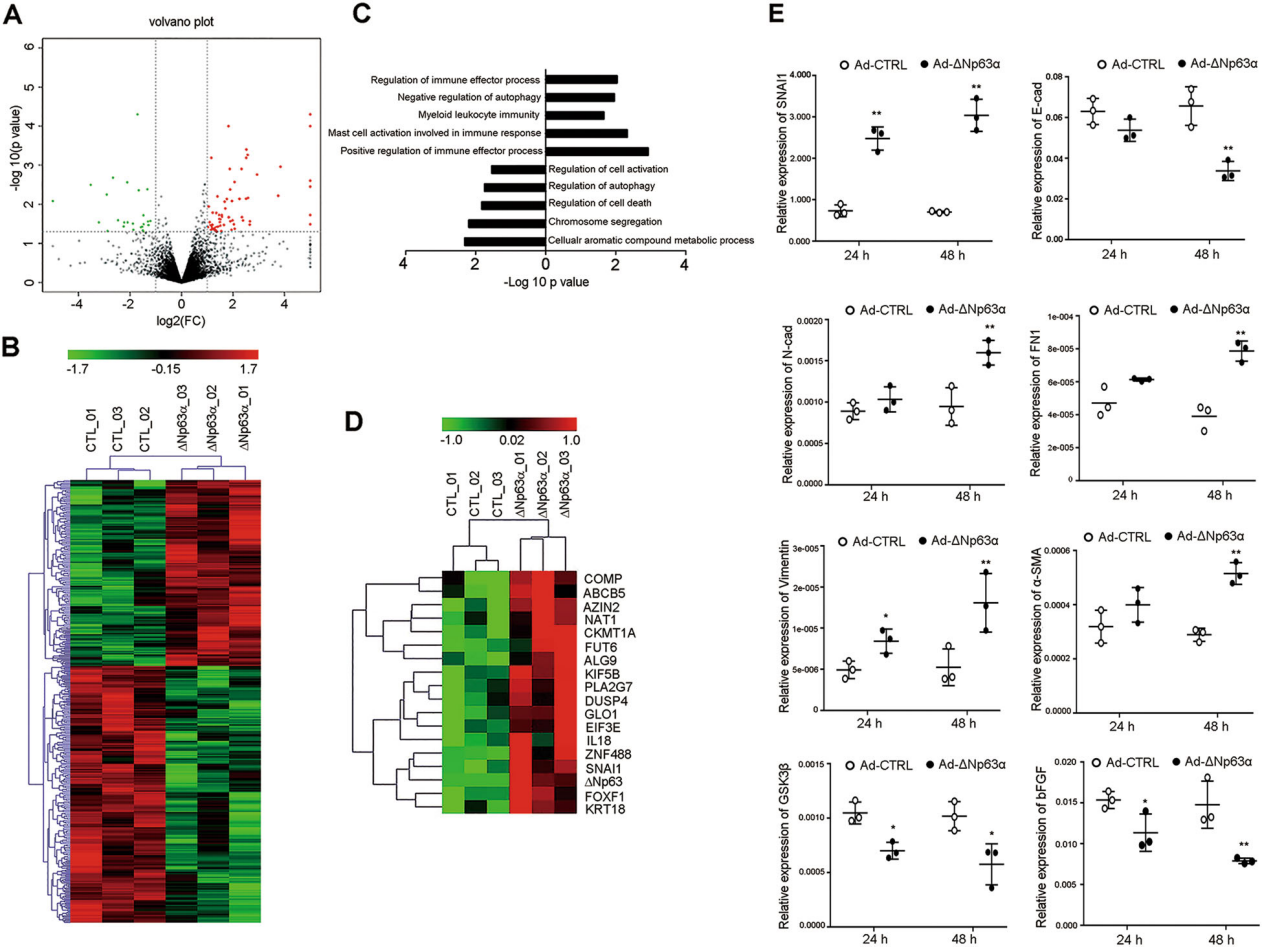
ΔNp63α enriches the transcription of genes that promote EMT in EECs. **a** Volcano plot representing DE genes in ΔNp63α (−) and ΔNp63α (+) EECs. Each plot represents a sample set. The statistical significance of all transcripts (*p* < 0.05 and fold change ≥ 2) was analyzed by Cuffdiff (v2.2.1). In the plot, green represents the downregulated transcripts and red represents the upregulated transcripts. **b** Heat map showing DE genes in ΔNp63α (−) and ΔNp63α (+) EECs. Each column represents a sample set. Each row represents an individual mRNA transcript. The statistical significance of all transcripts (*p* < 0.05 and fold change ≥ 2) was analyzed by Cuffdiff (v2.2.1). The heat map color spectrum corresponds to percentile ranks, indicating downregulated transcripts (green) and upregulated transcripts (red). **c** Major molecular functions of DE genes based on ontology analysis. GO analysis was performed using GO::Term Finder software. **d** Heat map of genes that promote EMT. Each column represents a sample set and each row represents an individual miRNA transcript. The heat map color spectrum corresponds to percentile ranks, showing downregulated transcripts (green) and upregulated transcripts (red). **e** qRT-PCR analysis of SNAI1, E-cadherin, N-cadherin, fibronectin (FN1), vimentin, α-SMA, GSK3β, and bFGF mRNA levels in EECs after incubation with Ad-ΔNp63α or Ad-CTL for 24 and 48 h (*n* = 3). *p < 0.05, and **p < 0.01.

As we found the ΔNp63α expression in EECs from the IUA patients (Fig. 1), we further examined whether ΔNp63α + lineage expansion induces EEC–EMT. Primary EECs (>95% purity identified with CK stain as shown in Supplementary Fig. 3) from normal controls were infected with either Ad-ΔNp63α or Ad-CTL. After 24 h, the RNA was extracted and subjected to RNA sequencing (RNA-seq) analysis. As shown in the volcano plot (Fig. 2a), 348 genes were differentially expressed in Ad-ΔNp63α- and Ad-CTL-infected EECs. The hierarchical clustering analysis of DEGs separated into two groups (Fig. 2b). ΔNp63α overexpression induced the upregulation of 146 genes and the downregulation of 202 genes in EECs (Supplementary Table 3). To verify the DEGs, we conducted qPCR to examine the mRNA levels of ten mostly upregulated and downregulated genes, and the results showed same differential expression patterns as those in RNA-seq (Supplementary Fig. 4).

Gene ontology (GO) enrichment analyses identified 7 biological processes from the upregulated genes and 20 biological processes from the downregulated genes (*p* < 0.05, Supplementary Table 4). Terms related to cellular aromatic compound metabolic processes, chromosome segregation, cell death, autophagy, and the regulation of cell activation and growth were significantly associated with the downregulated genes (Fig. 2c). Terms related to the positive regulation of immune effector processes, such as mast cell activation, leukocyte and myeloid leukocyte activation, and the inhibition of autophagy and the formation of the primary germ layer, were associated with the upregulated genes (Fig. 2c). Based on the GO terms from both upregulated and downregulated genes, ΔNp63α appears to promote the immune response and inflammation, to repress chromosome segregation and cell proliferation and development. All these biological processes have been demonstrated to be the driving factors in the induction of pathological EMT^20–23^.

During further screening of genes involved in EMT from ΔNp63α-induced EEC-transcriptomes, 18 genes that promote EMT were identified, including SNAI1, a key EMT driver. Hierarchical clustering analysis separated these 18 genes into two groups (Fig. 2d). By dissecting the SNAI1-mediated signaling pathway, we showed that SNAI1 was upregulated 24 h after Ad-ΔNp63α infection, while the SNAI1-mediated downregulation of CDH1 (E-cadherin) and upregulation of CDH2 (N-cadherin), α-SMA, VIM (vimentin), and FN (fibronectin) were not detected even 48 h after infection (Fig. 2e). However, GSK3β, a protein kinase that promotes SNAI1-ubiqutination and -degradation was downregulated both at 24 and 48 h. And bFGF expression was also downregulated both at 24 and 48 h (Fig. 2e). Thus, multiple molecules might be involved in SNAI1 activation.

### ΔNp63α induces the activation of SNAI1 signaling in EECs in vitro and in the endometria from IUA patients

The enhanced transcription of genes in EECs transfected with ΔNp63α (Fig. 2e) suggested that ΔNp63α induced a SNAI1-mediated phenotypic shift from an epithelial to a mesenchymal phenotype. Therefore, we examined the ΔNp63α-induced expression of the SNAI1 protein and SNAI1’s downstream proteins.

**Fig. 3:**
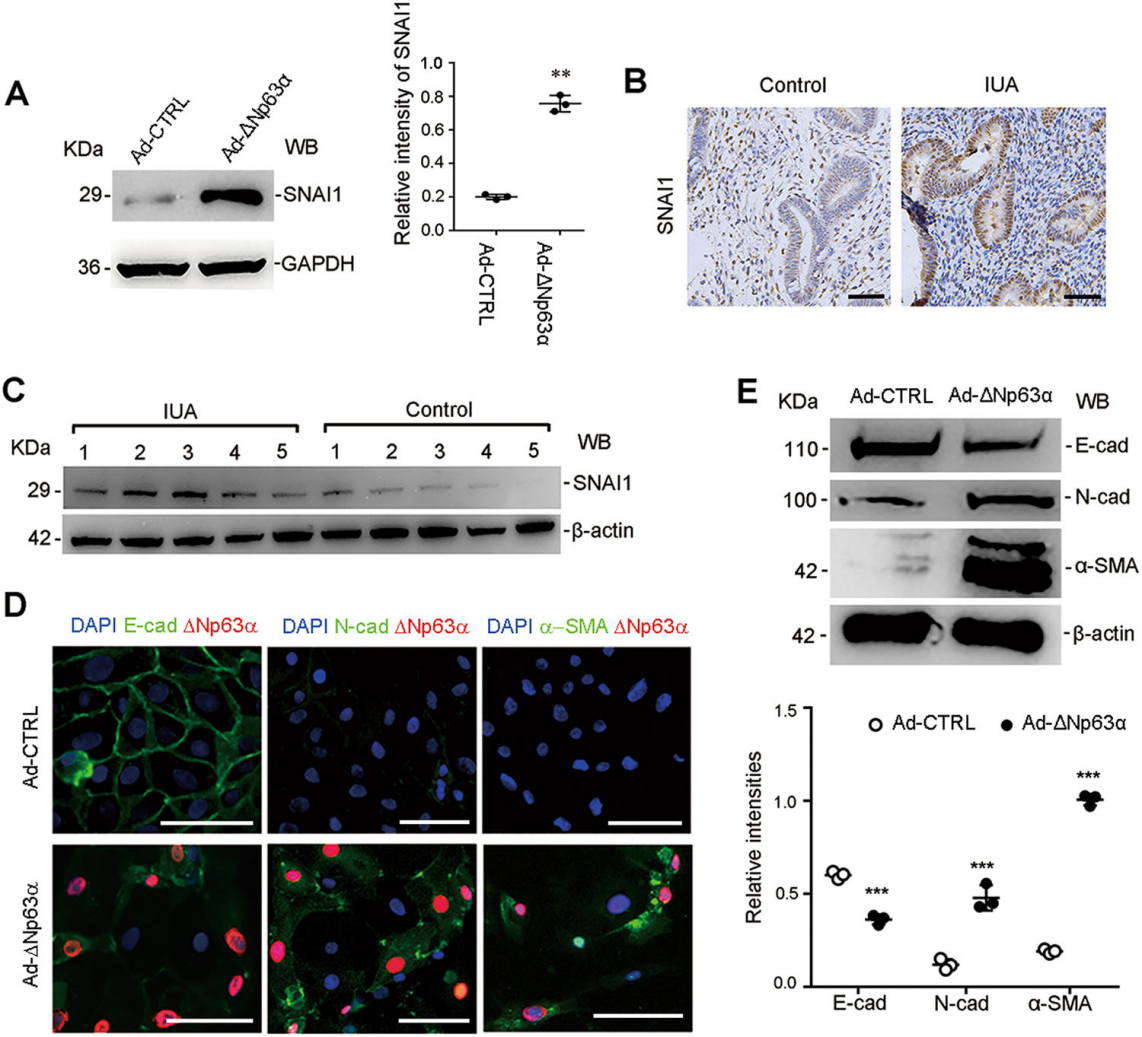
ΔNp63α promotes SNAI1 expression and EEC–EMT in vitro. **a** Protein levels of SNAI 1 were detected by western blotting in EECs after incubation with Ad-ΔNp63α or Ad-CTL for 48 h (*n* = 3). Relative band intensities were analyzed with Image J. **b** Representative images of SNAI1 immunostaining in normal (*n* = 30) and fibrotic endometria (*n* = 30). Scale bars, 50 μm. **c** Protein levels of SNAI 1 were detected by western blotting in endometria from normal control (*n* = 5) and IUA patients (*n* = 5). Relative band intensities were analyzed with ImageJ. **d** Representative images of ΔNp63α, E-cadherin, N-cadherin, and α-SMA immunofluorescence staining in EECs after incubation with Ad-ΔNp63α or Ad-CTL for 48 h (*n* = 3). Scale bars, 20 μm. **e** Protein levels of E-cadherin, N-cadherin, α-SMA, and β-actin were detected by western blotting in EECs after incubation with Ad-ΔNp63α or Ad-CTL for 48 h (*n* = 3). Relative band intensities were analyzed with ImageJ. Error bars, mean ± SD; **p < 0.01, and ***p < 0.001.

As shown in Fig. 3a, SNAI1 protein level in primary EECs infected with Ad-ΔNp63α had a fourfold increase compared to that in the cells infected with Ad-CTL. Critically, the SNAI1 protein in the endometria from IUA patients was also significantly upregulated in immunostaining (Fig. 3b) and immunoblotting (Fig. 3c). Meanwhile, E-cadherin, a key target of SNAI1, was downregulated in the immunostaining (Fig. 3d) and Western blotting showed that E-cadherin protein expression decreased to 59% of the control expression level (Fig. 3e). The ΔNp63α-induced upregulation of the N-cadherin and α-SMA proteins was further confirmed by immunostaining (Fig. 3d) and Western blotting displayed that N-cadherin and α-SMA proteins in the cells infected with Ad-ΔNp63 were increased by 4- and 5.2-fold respectively, compared to those in EECs infected with the control plasmid (Fig. 3e).

To further confirm the role of SNAI1 in ΔNp63α-induced EEC–EMT, we constructed a high expression vector of SNAI1 (pcDNA3.1-SNAI1). Then pcDNA3.1-SNAI1 or empty control vector was transfected into EECs with lipo3000. After 48 h, we detected the expression of E-cadherin, N-cadherin, α-SMA and SNAI1. The results showed that the SNAI1 protein was upregulated to 11.3-fold of the control level, E-cadherin was decreased by 52.8%, and N-cadherin and α-SMA were increased by a 1.85-fold and 3.07-fold, respectively (Supplementary Fig. 5A), which is in agreement with EEC–EMT. Then we transfected SNAI1 small interference sequences (siSNAI1) into ΔNp63α highly expressed EECs for 48 h. The results showed that siRNAs interference with SNAI1 reversed EEC–EMT (Supplementary Fig. 5B). These results further confirm that SNAI1 mediates the EEC–EMT induced by ΔNp63α.

### ΔNp63α induces the downregulation of GSK-3β (SNAI1 inhibitor) in EECs and in endometria of IUA patients

**Fig. 4:**
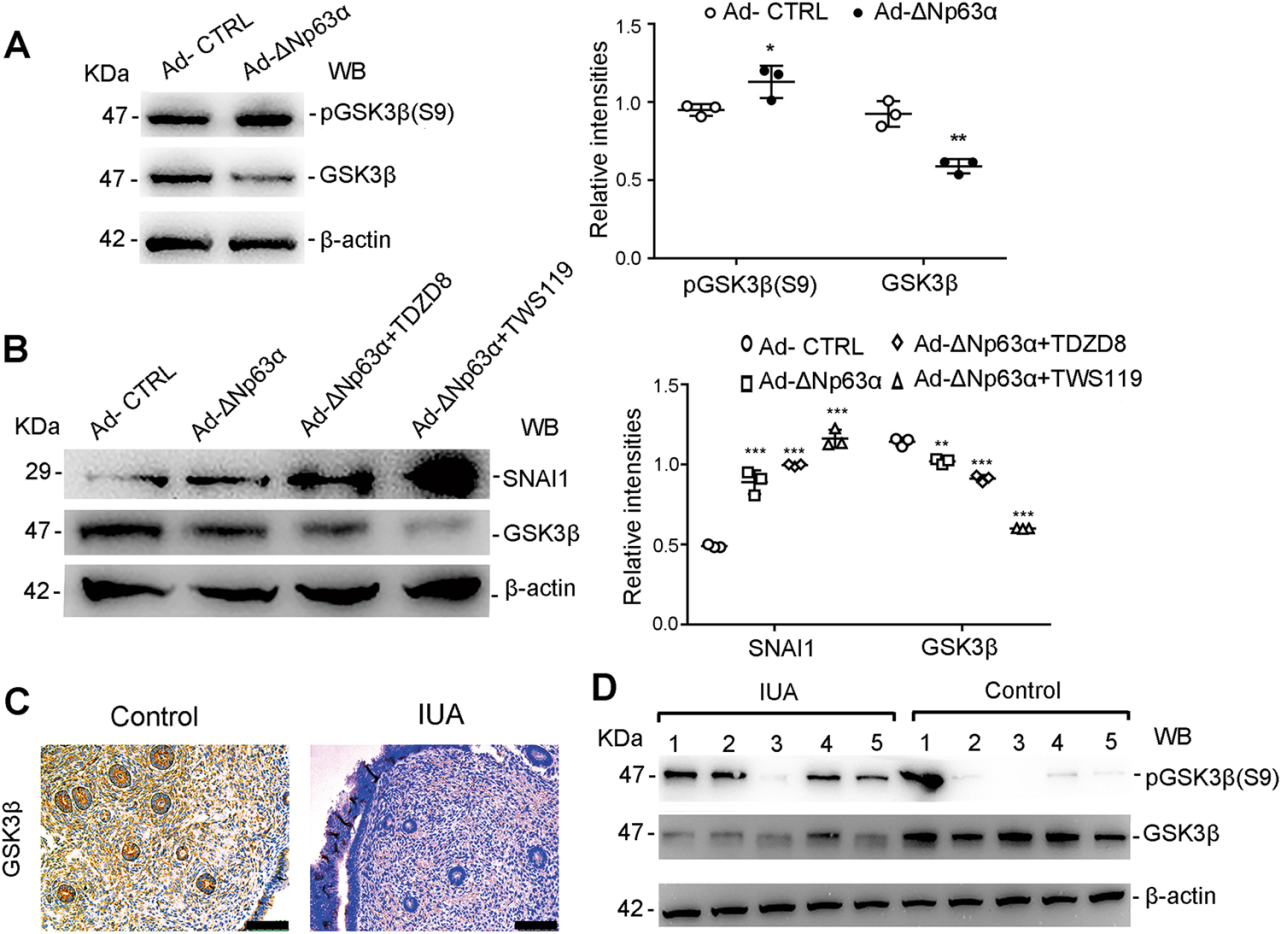
ΔNp63α induces the downregulation of the SNAI1 inhibitor, GSK3β in EECs. **a** Immunoblotting of pGSK3β(S9), GSK3β, and β-actin in EECs after incubation with Ad-ΔNp63α or Ad-CTL for 48 h (*n* = 3). Relative band intensities were analyzed with Image J. **b** Immunoblotting of SNAI 1, GSK3β, and β-actin in EECs after incubation with Ad-ΔNp63α or Ad-CTL and TDZD8 or TWS119 for 48 h (*n* = 3). Relative band intensities were analyzed with ImageJ. **c** Representative images of GSK3β immunostaining staining in normal (*n* = 30) and fibrotic endometria (*n* = 30). Scale bars, 10 μm. **d** Protein levels of pGSK3β(S9) and GSK3β were detected by western blotting in endometria from normal control (*n* = 5) and IUA patients (*n* = 5). Error bars, mean ± SD.; *p < 0.05, **p < 0.01, and ***p < 0.001.

The GSK3β-mediated degradation of SNAI1 is a crucial molecular mechanism that antagonizes EMT^24^. The inhibition of GSK-3β results in the upregulation of SNAI1 and the downregulation of E-cadherin, thus promoting EMT^25^. Since GSK-3β mRNA was downregulated in EECs infected with Ad-ΔNp63α (Fig. 2e), we further examined the GSK-3β protein level in Ad-ΔNp63α-infected EECs. Western blotting revealed that GSK-3β protein decreased to 49% of that in the cells infected with Ad-CTL and phospho-GSK3β Ser9 was upregulated by 1.2-fold (Fig. 4a), which is in accordance with the upregulation of SNAI1 protein (Fig. 3a). By treating Ad-ΔNp63α-infected EEC with TDZD8 and TWS119 (GSK-3β inhibitors), SNAI1 protein levels in Ad-ΔNp63α-infected EEC were 1.8-fold and 2.1-fold higher than those in Ad-CTL-infected cells in the presence of TDZD8 and TWS119 (Fig. 4b). Importantly, as showed in Fig. 4c, d, we found the decreased level of GSK-3β and the increased level of inactive form of GSK-3β, p-GSK-3β Ser9, in the endometria from IUA patients, which is consistent with the results obtained in the cultured cells (Fig. 4a).

### ΔNp63α induces DUSP4 downregulated GSK3β expression in EECs

Serine 9 of GSK3β can be phosphorylated by AKT and other kinases^26^, leading to GSK-3β inactivation. Thus, we examined the active form of AKT, pAKT Ser473, in Ad-p63α-infected EECs and in the endometria from IUA patients. As shown in Supplementary Fig. 6A, the expression of AKT and pAKT Ser473 did not significant change in Ad-p63α-infected EECs, and both proteins decreased in the endometria of IUA patients (Supplementary Fig. 6B). These results suggested that an alternative pathway existed for ΔNp63α-induced GSK-3β downregulation.

**Fig. 5:**
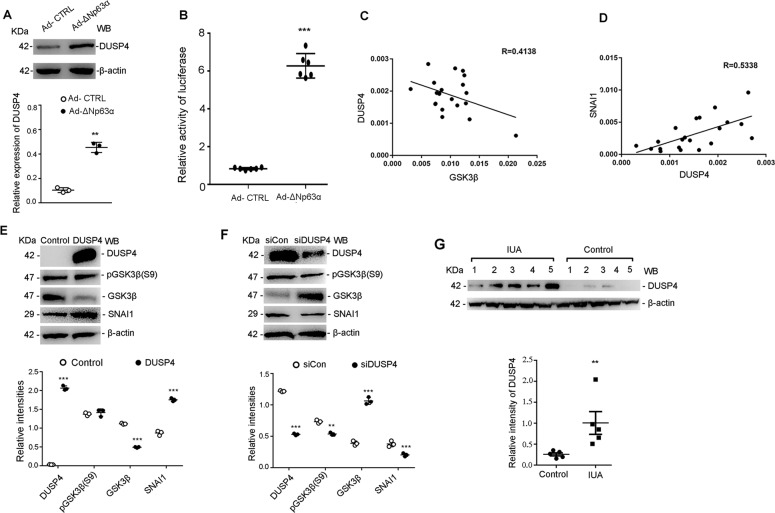
ΔNp63α induced DUSP4 downregulates GSK3β expression in EECs. **a** Protein level of DUSP4 was detected by western blotting in EECs after co-incubation with Ad-ΔNp63α or Ad-CTL for 48 h (*n* = 3). Relative band intensities were analyzed with Image J. **b** The fluorescence activity of DUSP4 promoter containing plasmid which was transfected into EECs with Ad-CTL or Ad-ΔNp63α for 12 h (*n* = 6). **c** The correlation of DUSP4 and GSK3β expression in endometria of IUAs (*n* = 21). Spearman’s correlation coefficient *R* = 0.4138, *p* < 0.05. **d** The correlation of DUSP4 and SNAI1 expression in endometria of IUAs (*n* = 21). Spearman’s correlation coefficient *R* = 0.5338, *p* < 0.05. **e** Immunoblotting of DUSP4, pGSK3β(S9), GSK3β, SNAI1, and β-actin in EECs after transfection with pCDNA3.1-DUSP4 or pCDNA3.1 for 24 h (*n* = 3). Relative band intensities were analyzed with ImageJ. **f** Immunoblotting of DUSP4, pGSK3β(S9), GSK3β, SNAI1 and β-actin in EECs after transfection with DUSP4-specific siRNAs for 24 h (*n* = 3). Relative intensities of bands were analyzed with Image J. **g** Immunoblotting of DUSP4 and β-actin in endometrium from IUA patients (*n* = 5) and normal control (*n* = 5). Relative band intensities were analyzed with ImageJ. Error bars, mean ± SD; *p < 0.05, **p < 0.01, and ***p < 0.001.

Then we looked back ΔNp63α-induced EEC-transcriptomes, and found that DUSP4 was one of the most upregulated gene, and it was known to be an important EMT promoting molecule^27,28^. To verify the promoting effect of ΔNp63α on the expression of DUSP4, we measured the protein level of DUSP4 in EECs infected with Ad-ΔNp63α, and found that DUSP4 protein was elevated by 1.5-fold (Fig. 5a). To investigate the role of ΔNp63α in promoting DUSP4 expression, we transfected Ad-ΔNp63α- or Ad-CTL-infected EECs with a fluorescence reporter plasmid containing DUSP4 promoter, and compared the fluorescence activity. The results are presented in Fig. 5b, in which fluorescent activity in EECs containing DUSP4 promoter was increased, indicating that ΔNp63α mainly promotes the expression of DUSP4 by acting on its promoter. We further analyzed the correlation between the expression of DUSP4 and GSK3β or SNAI1 respectively in endometria of IUA. The results showed that DUSP4 was negatively correlated with GSK3β (*R* = −0.4138) and positively correlated with SNAI1 (*R* = 0.5338) (Fig. 5c, d).

In order to further investigate the effect of DUSP4 on GSK3β, a DUSP4 expression plasmid (pCDNA3.1-DUSP4) and an empty plasmid (Control) were transfected into primary EECs. Interestingly, overexpressing DUSP4 also downregulated the GSK-3β protein to 32% of the control level. Accordingly, the expression of SNAI1 was 2.7-fold higher than that in the controls (Fig. 5e). However, DUSP4 had no effect on the expression of pGSK3β Ser9 (Fig. 5e). In contrast, by using short siRNAs to interfere with DUSP4 expression in ΔNp63α-overexpressing EECs, the results showed that the downregulation of the DUSP4 protein to 43% of the control level led to a 2.71-fold increase in the total GSK3β protein and a 47.4% and 27.4% decrease in SNAI1 and p-GSK-3β Ser9, respectively (Fig. 5f). Furthermore, we confirmed that DUSP4 protein was significantly increased in the endometrium from IUA patients compared with that from normal controls (Fig. 5g).

### bFGF reverses ΔNp63α induced EEC–EMT in vitro

**Fig. 6:**
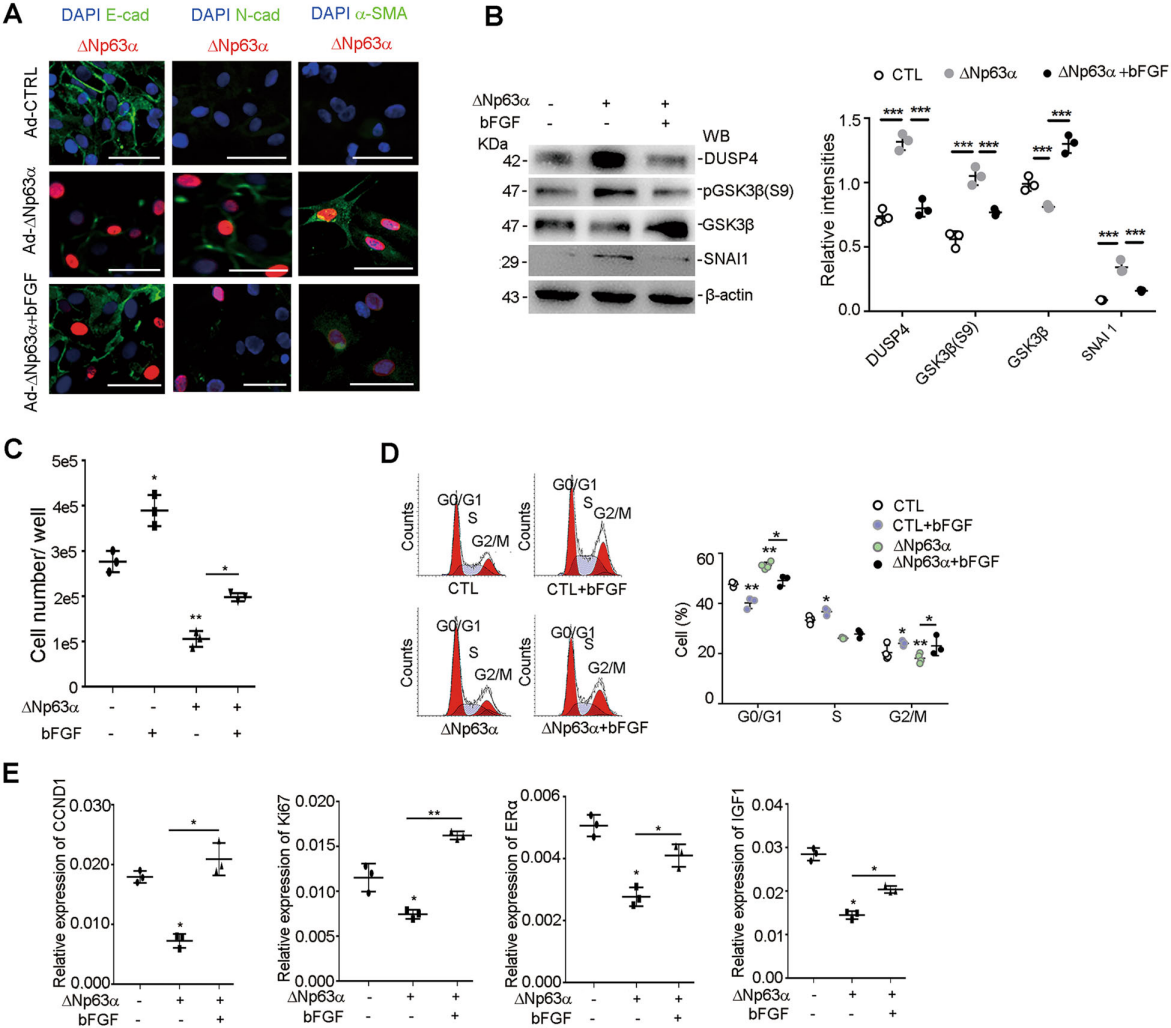
bFGF reverses ΔNp63α-induced partial EMT in EECs. **a** Representative image of immunofluorescence staining of ΔNp63α, E-cadherin, N-cadherin, and α-SMA in ΔNp63α-expressing EECs in the presence or absence of bFGF (*n* = 3). Scale bars, 25 μm. **b** Immunoblotting of DUSP4, pGSK3β(S9), GSK3β, SNAI1, and β-actin in EECs after 48 h of infection with Ad-ΔNp63α or Ad-CTL with or without bFGF (*n* = 3). Relative band intensities were analyzed with Image J. **c** The number of EECs was counted after infection with Ad-ΔNp63α or Ad-CTL for 48 h (*n* = 3). **d** EECs were infected with Ad-ΔNp63α or Ad-CTL. After 48 h of infection, cells were subsequently assayed for their DNA content by a flow cytometry assay (*n* = 3). Cell cycle distribution images are shown, and the percentage of cells in different phases of the cell cycle was statistically analyzed. **e** EECs were infected with Ad-ΔNp63α or Ad-CTL. After 24 h, the mRNA levels of CCND1, Ki67, ERα and IGF1 were examined by qRT-PCR (*n* = 3). Error bars, mean ± SD; *p < 0.05, and **p < 0.01.

The data in our present study showed that the pathogenesis of IUA involves EEC–EMT. Previously, we found the proliferation of endometrium is suppressed in ΔNp63α overexpressed endometrium^8^, and bFGF promotes the proliferation of endometrium and functionally reconstructs endometrium in rats and patients^29,30^. Therefore, we tried to study the relationship between bFGF and ΔNp63α-induced EEC–EMT. In the presence of bFGF (0–20 ng/ml), ΔNp63α-infected EECs showed dose-dependent growth, with 10 ng/ml as an optimal concentration (Supplementary Fig. 7A). The expression of ΔNp63α, E-cadherin, N-cadherin, α-SMA, DUSP4, SNAI1, and GSK3β was detected in ΔNp63α-infected EECs in the presence of bFGF. The results showed that ΔNp63α expression was inhibited by bFGF and the ΔNp63α-induced downregulation of E-cadherin and upregulation of N-cadherin and α-SMA were reversed by bFGF (Fig. 6a). Furthermore, the ΔNp63α-induced upregulation of DUSP4 and SNAI1 and downregulation of GSK3β were also reversed by bFGF treatment in EECs (Fig. 6b). Further tests with cytometry showed that the cell cycle switched from G0/G1 to S/G2 phase in the presence of bFGF (Fig. 6c, d), with the transcriptional upregulation of CCND1, Ki67, ERα and IGF1 (Fig. 6e) and a slight downregulation of cell apoptosis (Supplementary Fig. 7B), indicating that bFGF reversed Ad-ΔNp63α-induced repression of endometrial proliferation and differentiation.

### bFGF functionally regenerates endometrium in mice with IUA-like endometrial lesions

**Fig. 7:**
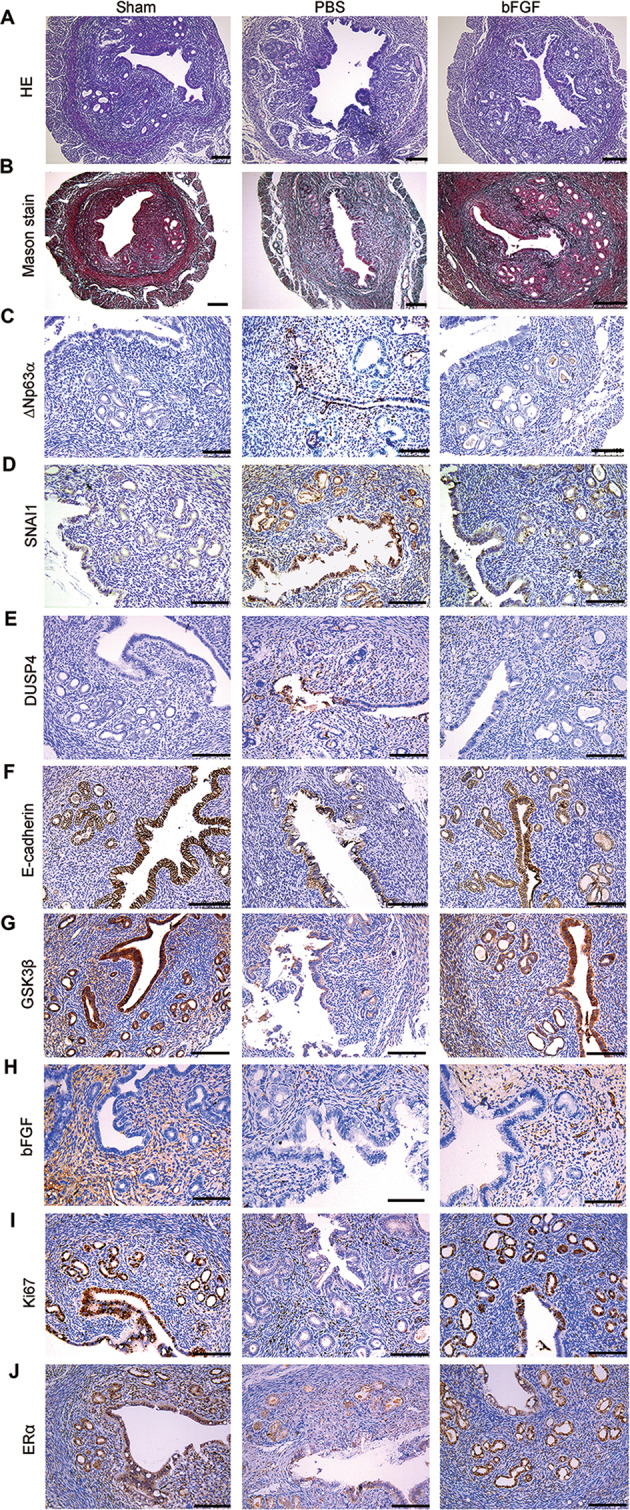
bFGF attenuates endometrial fibrosis in mice. Representative images of **a** HE, **b** Mason staining, **c** ΔNp63α, **d** SNAI1, **e** DUSP4, **f** E-cadherin, **g** GSK3β, **h** bFGF, **i** Ki67, and **j** ERα in endometrial biopsies of sham (*n* = 6), endometrial fibrosis model mice with PBS treatment (*n* = 5) and endometrial fibrosis model mice with bFGF treatment (*n* = 5). Scale bars, 100 μm.

Trauma and inflammation are key factors leading to IUA. In order to generate an IUA mouse model, we injured endometria by mechanical injury and administration of lipopolysaccharide (LPS) in uterine cavity as previously reported^17^. Compared to the sham mice, the mouse model showed the reduction of endometrial glands (Fig. 7a), the accumulation of collagen protein with positive Masson staining (Fig. 7b), the increment of ΔNp63α protein (Fig. 7c), SNAI1 (Fig. 7d), and DUSP4 (Fig. 7e), and the reduction of E-cadherin (Fig. 7f), GSK3β (Fig. 7g), bFGF (Fig. 7h), Ki67 (Fig. 7i), and ERα (Fig. 7j) in the endometria of these mice. These results indicated that the mouse model simulated the endometrial lesions in IUA patients.

Compared to the mouse model treated with PBS, the mice treated with bFGF had functionally regenerated endometrium, with increased endometrial glands (Fig. 7a), alleviated collagen deposition (Fig. 7b), decrement of ΔNp63α protein (Fig. 7c), SNAI1 (Fig. 7d), and DUSP4 (Fig. 7e), and increment of E-cadherin (Fig. 7f), GSK3β (Fig. 7g), bFGF (Fig. 7h), Ki67 (Fig. 7i), and ERα (Fig. 7j) in the endometria of the bFGF- treated mice. The changes in the mRNA levels of these molecules were similar to proteins changes as shown in Supplementary Fig. 8. These results indicated that bFGF reverted the phenotype of IUA-like mouse model.

## Discussion

IUA is characterized with endometrial fibrosis, yet the pathological process is mostly unknown. Here, we demonstrated that EEC–EMT is actively involved in the pathogenesis of endometrial fibrosis. We found for the first time that ΔNp63α drives EEC–EMT by promoting the activity of the SNAI1 pathway and that bFGF reverses ΔNp63α-induced EEC–EMT. The results in the present study provide the potential therapeutic target for IUA patients.

ΔNp63α is the predominantly expressed protein isoform of p63 in epithelial cells, which is well-known for its function in maintaining squamous epithelial cell integrity and as an epithelial progenitor cell marker, ΔNp63α regulated keratinocytes regeneration^31,32^. Previous studies showed the expression of p63 turns off in endometrium after the fusion of bilateral paramesonephric ducts^10^. In the present study, we found that EECs ectopically expressed ΔNp63α in patients with IUA, which was further confirmed by the co-localization staining of ΔNp63α with CK and E-cadherin in EECs. Several studies indicated that p63 expression is related to tissue fibrosis, in which EMT plays a critical role. In early stage of idiopathic pulmonary fibrosis, increased expression of ΔNp63α in bronchopulmonary epithelium can promote EMT, resulting in lung fibrosis^33^. High expression of ΔNp63α in sublingual gland epithelial cells can also lead to EMT and promote fibrosis^34–37^.

In the present study, we found that the expression of ΔNp63α in EECs causes type 2 EMT. This is supported by the hybrid phenotype with both epithelial and mesenchymal traits in upregulated ΔNp63α EECs in severe IUA patients, which was validated by the downregulation of E-cadherin (epithelial marker) and the upregulation of N-cadherin, Vimentin, and α-SMA (mesenchymal markers) in ΔNp63α overexpressed EECs and IUA-like mouse model.

In searching how ΔNp63α expression in EECs causes type 2 EMT, we found that ΔNp63α upregulates the transcription factor SNAI1, and downregulates GSK-3β. SNAI1 is an important inducer of EMT^38^. GSK-3β is an inhibitory kinase of SNAI1 and increment of GSK-3β in the cells can block EMT process by enhancing degrading SNAI1^25^. In addition, we also found that upregulation of ΔNp63α increases DUSP4, and the DUSP4 expression is negatively correlated with the GSK-3β level in EECs. The overexpression of DUSP4 in EECs mimicked ΔNp63α-induced EEC–EMT, and inhibition of DUSP4 reversed EEC–EMT, indicating the important role of DUSP4 in EEC–EMT. Nevertheless, the detailed molecular process requires further studies.

Of the 69 patients initially enrolled, 43.5% (30/69) had detectable mRNA and protein of ΔNp63, 30.4% (21/69) had no detectable mRNA and protein, and some patients just showed detectable mRNA or protein. There are several possibilities for the disagreement in the detection of mRNA and protein in these patients. The undetectability of mRNA and protein in 21 patients may reflect that there is no expression of ΔNp63α, the expression level is too low to be detected, or the expression is focalized so that the sampling missed the foci that express ΔNp63α. Probably, because of the focalized expression of ΔNp63α, the results of mRNA and IHC were not in agreement in some patients.

bFGF functions to enhance the tissue repair. Recently, in vitro study showed that bFGF inhibits the transition of fibroblast to myofibroblast^39^. In the human, we previously demonstrated that bFGF has the activity against fibrosis in fibrotic endometria^29^. In the present study, the administration of bFGF in IUA-like mice improved the endometrial fibrosis while ΔNp63α and SNAI1 levels in EECs were reduced, and E-cadherin was increased and N-cadherin and a-SMA were decreased. These results provided solid evidence that ΔNp63α plays an important role in the pathogenesis of IUA and that ΔNp63α may serve as a potential target for IUA therapy.

## Supplementary information

Supplementary Materials and methods

Supplementary figure legends and Tables

Supp. Figure 1

Supp. Figure 2

Supp. Figure 3

Supp. Figure 5

Supp. Figure 4

Supp. Figure 6

Supp. Figure 7

Supp. Figure 8
