# Supplementary material for: ΔNp63α-induced DUSP4/GSK3β/SNAI1 pathway in epithelial cells drives endometrial fibrosis
Source: Cell Death Dis. 2020 Jun 11;11(6):449. doi: 10.1038/s41419-020-2666-y (PMC7289806; doi:10.1038/s41419-020-2666-y)
Supplement: Supplementary file 1 — Supplementary Materials and methods [file 41419_2020_2666_MOESM1_ESM.docx]

**Materials and methods**

**Endometrial biopsy**

For hysteroscopy examinations, IUA patients and normal control women were scheduled in the late proliferative phase of the menstrual cycle, based on the ultrasonography (follicle size 15-18 mm) and a low level of serum progesterone. Endometrial samples were taken from two sites in the uterine body or fundus that showed the most severe adhesion. For normal controls, samples of endometria were taken in the uterine body and fundus. Approximately half of the tissue samples were stored in liquid nitrogen and remained half were fixed with formalin and used for further experiments as described below.

**Immunolocalization**

Endometrial tissues were fixed in 10% formaldehyde for 8 hours at 4°C, transferred to a tissue processor (Leica ASP300 S, Wetzlar, Germany) for dehydration and wax infiltration, and embedded in paraffin. Paraffin blocks were cut into 2-μm-thick slices. The endogenous peroxidase activity was blocked with 3% H_2_O_2_. After heat-mediated antigen retrieval, slides were incubated with 10% serum from the species that generated the secondary antibody to block nonspecific binding. Tissue sections were labeled with primary antibodies overnight at 4°C. Negative controls were generated using pre-immune rabbit or mouse IgG or by omitting the primary antibodies. After incubation with HRP-conjugated secondary antibodies, sections were exposed to 3’3-diaminobenzidine to visualize the antigen signals. Sections were then counterstained with hematoxylin and viewed under a microscope (DMi8, Leica, Wetzlar, Germany). A whole tissue slice was scanned with LAS X software (Leica, Germany). Then immunohistochemical staining was quantified by mean optical density using 2D analysis by LAS X software (Leica, Germany). The positive staining was confirmed in a blinded manner by two independent observers. The procedure of tissue immunofluorescence before secondary antibodies incubation was the same as that of immunohistochemistry. In tissue immunofluorescence, the secondary antibodies were labeled by fluorescence. Then samples were examined with a microscope (DMi8, Leica, Wetzlar, Germany) equipped with filters to selectively view the rhodamine and fluorescein fluorescence with no cross-contamination. For cell immunofluorescence, isolated EECs that had been plated on Matrigel-coated coverslips for varying times were fixed in 3% paraformaldehyde for 10 minutes and permeabilized with cold methanol. Fixed cells were stained with a primary antibody for varying incubation periods (1 hour to overnight), washed, and incubated with secondary antibodies conjugated to fluorescein or rhodamine. Secondary antibodies were cross-absorbed against nonimmune IgG of another species to eliminate cross-reactivity. Samples were viewed with a microscope (DMi8) equipped with filters to selectively view the rhodamine and fluorescein fluorescence with no cross-contamination.

**EEC isolation and culture**

The isolation and culture of EECs were performed as described [8]. Briefly, normal endometrial tissues were digested with a mixture of collagenase type I, hyaluronidase and DNase, and were then size-fractionated with a 40-μm cell strainer (BD Biosciences, San Jose, CA, USA) to separate the fragments of the endometrial glands from the stromal cells. EECs from the glands were plated on Matrigel-coated dishes and cultured with defined keratinocyte serum-free medium (KSFM; Gibco, Massachusetts, USA). The cell purity was verified by cytokeratin (CK) staining. Freshly isolated EECs were immediately used.

**Preparation of recombinant ΔNp63α adenovirus and EECs infection**

ΔNp63α adenovirus and control adenovirus were constructed as described elsewhere [8]. The open reading frame (GenBank: AF075431.1) and a partial 3′UTR of Homo sapiens ΔNp63α were cloned into a DC315-3FLAG-SV40-EGFP vector and inserted into a shuttle plasmid. Next, the shuttle plasmid and adenoviral backbone plasmid were used to co-transfect HEK-293A cells to produce the recombinant adenoviral vector, Ad-ΔNp63α. The same protocol was used to generate the control virus, Ad-CTL, using the pDC315-3FLAG-SV40-EGFP vector without the ΔNp63α insert (GeneChem, Shanghai, China). The efficiency of different titers of Ad-CTL or Ad-ΔNp63α were verified as described in our previous study [8] and in this study 1×10^5^ primary EECs were infected with 1×10^7^ PFU/ml adenovirus.

**Quantitative real-time PCR and western blot analysis**

RNA isolation, quantitative real-time PCR (qRT-PCR) and western blotting were performed as described [8]. All experiments were repeated at least three times.

**Cell cycle analysis**

Cell cycle analysis was performed to evaluate the effects of bFGF on the ΔNp63α positive or negative EECs. Briefly, EECs were infected with Ad-CTL or Ad-ΔNp63α for 12 hours, then treated with 10ng/ml bFGF for another 48 hours.The cells were then fixed in cold 70% ethanol and incubated overnight at 4°C. After washing twice with PBS, the cells were incubated with 50 μg/ mL PI and 20 μg/ mL RNase A for 30 min at room temperature and detected by FACS. The data were analyzed with ModFit 3.0 software. All experiments were repeated (n=3).

**Luciferase assays**

DUSP4 promoter (NM_001394, nt-1222 to nt+16) was synthesized and inserted into pGL3-pro-luciferase reporter (Promega). For luciferase assays, cells were plated in 24-well plates at a density of 1.5×104 cells per well. Transient transfection was performed using lipo3000 (Invitrogen). Each well received 250 ng of a pGL3-pro-luciferase reporter and 5 ng of a Renilla luciferase reporter (pRL-tk) from Promega. At the same time, Ad-CTL or Ad-ΔNp63α were added into above transfected cells. After 12 hours, the cells were harvested using Promega’s Passive Lysis buffer. Luciferase and Renilla luciferase activities were determined using Promega’s Dual Luciferase assay in a Plate Chameleon luminometer (BioScan, Washington, DC). Firefly luciferase was normalized by Renilla luciferase to correct for transfection efficiency.

**siRNA knockdown**

For silencing of SNAI 1 and DUSP4, siRNA knockdown was performed in EECs using siRNAs obtained from Ribobio (Guangzhou, China). The cells were transfected with lipo3000 according to the manufacturer’s protocol. The sequences of siRNA as follows: siDUSP4-1: GAAGGACACTATCAGTACA; siDUSP4-2: GCATCACGGCTCTGTTGAA; siDUSP4-3: CCTTCGAGTTCGTTAAGCA; siSNAI1-1: CAAGGAATACCTCAGCCTG; siSNAI1-2: GATGCACATCCGAAGCCAC; siSNAI1-3: GGCCTTCAACTGCAAATAC
