# Supplementary material for: ΔNp63α-induced DUSP4/GSK3β/SNAI1 pathway in epithelial cells drives endometrial fibrosis
Source: Cell Death Dis. 2020 Jun 11;11(6):449. doi: 10.1038/s41419-020-2666-y (PMC7289806; doi:10.1038/s41419-020-2666-y)
Supplement: Supplementary file 2 — Supplementary figure legends and Tables [file 41419_2020_2666_MOESM2_ESM.docx]

**Fig. S1 ΔNp63α and EEC-EMT markers are upregulated in endometrial biopsies from IUA patients.**

**a** Representative image of ΔNp63α immunostaining in endometrial biopsies of normal (n=30) and fibrotic endometria (n=30). **b** Representative images of Masson staining in normal (n=30) and fibrotic endometria (n=30). **c** Representative images of CK, E-cadherin, N-cadherin, Vimentin and α-SMA immunostaining in endometrial biopsies of normal (n=30) and fibrotic endometria (n=30). Scale bars, 50 μm.

**Fig. S2 ΔNp63α and cytokeratin (CK) staining in endometrial biopsies.**

Representative images of colocation of ΔNp63α with cytokeratin (CK) in endometrial biopsies of IUAs. Scale bars, 25 μm.

**Fig. S3 CK staining of primary EECs.** Representative image of cytokeratin (CK) staining in primary EECs. Scale bars, 25 μm.

**Fig. S4 Verification of differentially expressed genes by qPCR.** Several differentially expressed genes in ΔNp63α (-) and ΔNp63α (+) EECs were detected by qPCR

**Fig. S5 SNAI1 mediates the effects of ΔNp63****α on EEC-EMT. a** Immunoblotting of E-cad, N-cad, α-SMA and Snai1and β-actin in EECs after transfection with pcDNA3.1-snai1 or empty control was for 48 hours (n=3). Relative band intensities were analyzed with Image J. **b** Snai1 small interference sequences (siSNAI1) were transfected into ΔNp63α highly expressed EECs for 48 hours. Then the expression of E-cad, N-cad, α-SMA, ΔNp63α, Snai1and β-actin was detected. Relative band intensities were analyzed with Image J. * p < 0.05, and ** p < 0.01.

**Fig. S6 The effects of ΔNp63α on AKT activation in EECs. a** Immunoblotting of pAKT (S473), AKT and β-actin in EECs after incubation with Ad-ΔNp63α or Ad-CTL for 48 hours (n=3). Relative band intensities were analyzed with Image J. **b** Protein levels of pAKT (S473), AKT and β-actin were detected by western blotting in endometria from normal control (n=5) and IUA patients (n=5). Relative band intensities were analyzed with Image J.

**Fig. S7 The effects of bFGF on the proliferation and apoptosis of ΔNp63α (-) or ΔNp63α (+) EECs. a** Cell proliferation activity of ΔNp63α (-) or ΔNp63α (+) EECs was detected after treatment with different concentrations of bFGF for 48 hours. **b** Cell apoptosis of ΔNp63α (-) or ΔNp63α (+) EECs was detected after treatment with 10ng/ml bFGF for 48 hours. Error bars, mean ± s.d.; * p < 0.05, ** p < 0.01, and *** p < 0.001.

**Fig. S8 Detection of mRNA levels of ΔNp63α-induced genes in IUA-like mice.** The mRNA levels of ΔNp63α, E-cadherin (CDH1), N-cadherin (CDH2), α-SMA, DUSP4, GSK3B and SNAI1 in endometrial biopsies of Sham (n=6), endometrial fibrosis model mice with PBS (n=5) and endometrial fibrosis model mice with bFGF treatment (n=5) were examined by qRT-PCR.

**Table S1 Information of patients with intrauterine adhesions (IUA)**

| **Items** | **Control (n = 30)** | **IUA (n = 30)** | **P value** |
| --- | --- | --- | --- |
| Age (year) | 30.1 ±1.03 | 30.8 ± 1.60 | > 0.05 |
| Duration of infertility (year) | 1.9 ± 0.73 | 6.56 ± 3.69 | < 0.05 |
| AFS IUA score under hysteroscopy* | 0 | 10.15 ± 1.66 | < 0.05 |
| Endometrial thickness (mm)  (late-proliferative phase) | 9.04 ± 1.78 | 5.21 ± 1.46 | < 0.05 |

***** AFS, American Fertility Society. A score ≥8 indicates severe intrauterine adhesions.

**Table S2 Reagents, sources, concentration and applications**

| **Reagents** | **Source (catalogue numbers)** | **Concentration** | **Application** |
| --- | --- | --- | --- |
| Mouse anti-ΔNp63 | Zytomed Systems (MSG097) | 1:100 | IHC-P/IF |
| E-cadherin | Abcam (ab1416) | 1:400 | IHC-P/IF/WB |
| N-cadherin | Abcam (ab18203) | 1:400 | IHC-P/IF/WB |
| Alpha-SMA | Abcam (ab32575) | 1:400 | IHC-P/IF/WB |
| Phospho-GSK-3β (Ser9) (D85E12) | Cell Signaling (5558) | 1:1000 | WB |
| GSK-3β (27C10) Rabbit mAb | Cell Signaling  (9315) | 1:1000 | IHC-P/WB |
| DUSP4/MKP2 (D9A5) Rabbit mAb | Cell Signaling (5149) | 1:1000 | WB |
| Anti-DUSP4 | Abcam (ab72593) | 1:300 | IHC-P |
| Anti-Ki67 | Abcam (ab15580) | 1:400 | IHC-P |
| Anti-Estrogen Receptor alpha antibody [E115] | Abcam (ab32063) | 1:400 | IHC-P |
| Rabbit anti-Snail | Cell Signaling (3879) | 1:1000 | WB |
| Polyclonal anti-ΔNp63 | Millipore (ABS552) | 2.0μg/ml | WB |
| Rabbit anti-p-AKT(Ser473) | Abclonal (AP0140) | 1:1000 | WB |
| Mouse anti-total-AKT | Cell Signaling (2920) | 1:1000 | WB |
| Anti-FGF-2 (C-2) | Santa Cruz  (sc-74412) | 1:400 | IHC-P |
| β-Actin (8H10D10) Mouse | Cell Signaling (3700) | 1:1000 | WB |

| **Table S3 348 differentially expressed genes** | | | | | |
| --- | --- | --- | --- | --- | --- |
| **Up-regulated genes** | | | **Down-regulated genes** | | |
| **Gene Name** | **Folds change** | **P value** | **Gene Name** | **Folds change** | **P value** |
| TP63 | 945.43138 | 0.01432928 | SNRPB | -9.6537333 | 0.02546847 |
| KRT18 | 63.8283333 | 0.0325239 | TFRC | -8.3819 | 0.03363391 |
| IL18 | 19.8471333 | 0.04112582 | FAM96A | -6.6919667 | 0.03280818 |
| CYP51A1 | 17.5963333 | 0.03867901 | AKR1B1 | -6.5521333 | 0.00149132 |
| EIF3E | 15.2486667 | 0.04825563 | TNS3 | -6.2856 | 0.01206047 |
| ALCAM | 14.6633333 | 0.00113401 | PEPD | -5.8603 | 0.01968283 |
| SERPINB2 | 12.1997 | 0.03614875 | ATP6V1G1 | -5.793 | 0.01549324 |
| GLO1 | 11.4993333 | 0.02424686 | CEBPB | -5.7488333 | 0.02689904 |
| HADHA | 10.4830333 | 0.01563033 | PNP | -5.2556333 | 0.02073154 |
| C14orf166 | 9.42726667 | 0.0495877 | HMGB1P6 | -5.2337667 | 0.04848633 |
| MRPL27 | 7.26423333 | 0.01502563 | KANSL1 | -5.0516 | 0.04406912 |
| TXN2 | 6.52646667 | 0.031583 | TTC39C | -4.8104667 | 0.03113544 |
| JKAMP | 6.45133333 | 0.00748735 | ICAM1 | -4.488 | 0.00914394 |
| KIF5B | 6.32233333 | 0.01371426 | MCRS1 | -4.488 | 0.04969767 |
| YIPF3 | 6.23053333 | 0.02159986 | PRRC2A | -3.9164333 | 0.02113469 |
| EDN2 | 6.1178 | 0.00699102 | ELP2 | -3.7100333 | 0.0470516 |
| NAP1L1 | 5.774 | 0.04406222 | TEAD2 | -3.695 | 0.04678633 |
| DUSP4 | 5.53013333 | 0.04745071 | ITPKC | -3.5361667 | 0.01526038 |
| SLC25A24 | 4.1502 | 0.01809304 | RAB12 | -3.2574667 | 0.01270081 |
| SPDL1 | 3.96086667 | 0.00586323 | SLC25A11 | -3.2033 | 0.03434944 |
| NBEAL1 | 3.9153 | 0.02957943 | CISD2 | -3.1715333 | 0.03823863 |
| SNAPC1 | 3.87416333 | 0.01368873 | ETNK1 | -3.1645 | 0.03906314 |
| GCN1 | 3.75573333 | 0.04120335 | SPATS2 | -3.1632733 | 0.02839243 |
| TXNDC9 | 3.70206667 | 0.0390703 | UNG | -2.8512867 | 0.02830313 |
| TIGAR | 3.37843333 | 0.00367774 | SRSF4 | -2.7803667 | 0.03956783 |
| RAB10 | 3.3245 | 0.04540862 | MED28 | -2.7608167 | 0.01848304 |
| ERCC5 | 2.81886667 | 0.03738087 | UCK2 | -2.7359333 | 0.03374443 |
| IMMT | 2.7931 | 0.00593684 | PARP1 | -2.7241 | 0.04909387 |
| SRP19 | 2.73496667 | 0.01665176 | SEH1L | -2.638 | 0.0354222 |
| MRPL30 | 2.70733333 | 0.00773238 | GLG1 | -2.5409 | 0.01987001 |
| DMXL1 | 2.6823 | 0.01202691 | SPX | -2.5153567 | 0.02943218 |
| SUCLG1 | 2.67496667 | 0.02942707 | UBE2V2 | -2.4901333 | 0.03225763 |
| MAP4K5 | 2.66716667 | 0.03857708 | ARHGAP26 | -2.4772967 | 0.04181233 |
| NMD3 | 2.66343333 | 0.03466271 | ABR | -2.4686333 | 0.0312868 |
| IFRD1 | 2.64706667 | 0.04908431 | CRLS1 | -2.431 | 0.01981873 |
| ZNF28 | 2.49086667 | 0.02718743 | PDSS2 | -2.3924967 | 0.00994944 |
| KNTC1 | 2.43096667 | 0.04627789 | RERE | -2.3117667 | 0.049392 |
| STAM | 2.2849 | 0.03998331 | GOLGA4 | -2.2157 | 0.01618441 |
| PPIL3 | 2.19319667 | 0.01070296 | ATP6V0A2 | -2.0859433 | 0.01400146 |
| CEP70 | 2.18686667 | 7.73E-05 | SLC43A3 | -2.0476 | 0.01893503 |
| MRPL9 | 2.16826667 | 0.01146932 | ADO | -2.023 | 0.00497876 |
| ALG9 | 2.0893 | 0.04308388 | BSDC1 | -1.9419333 | 0.02459167 |
| BAZ2B | 2.03646667 | 0.02894444 | INSR | -1.87056 | 0.03194086 |
| BLCAP | 2.03456667 | 0.02103101 | XPO5 | -1.8409333 | 0.04171155 |
| RGS20 | 2.02171 | 0.02222756 | WDR48 | -1.7704667 | 0.00837059 |
| VILL | 1.95091 | 0.00541877 | SLC15A4 | -1.7660667 | 0.04159261 |
| NAT1 | 1.90484 | 0.0390496 | TRMT1 | -1.72158 | 0.02840684 |
| MRPL35 | 1.8771 | 0.03404562 | SUPV3L1 | -1.6954333 | 0.03154312 |
| EDEM3 | 1.8633 | 0.03485623 | MAFG | -1.6841667 | 0.03354549 |
| SERPINB5 | 1.81667667 | 0.01331936 | GIT1 | -1.6837767 | 0.01529194 |
| SNX16 | 1.7426 | 0.02548688 | LMAN2L | -1.6804633 | 0.03277429 |
| TTC17 | 1.7346 | 0.00250176 | ARL15 | -1.6306533 | 0.02202354 |
| PPP5C | 1.68466667 | 0.03592626 | UBL4A | -1.6187333 | 0.00183536 |
| POU2F3 | 1.68083333 | 0.03666701 | DDX50 | -1.6186467 | 0.0351954 |
| RAET1L | 1.64686033 | 0.01478079 | DPH5 | -1.5928 | 0.0476516 |
| SLC35D1 | 1.64426667 | 0.01331185 | ITPRIP | -1.5705133 | 0.01431698 |
| RUBCN | 1.546 | 0.03429697 | SH3BP2 | -1.53016 | 0.0168967 |
| CKMT1B | 1.52182667 | 0.00169565 | CASC3 | -1.4798667 | 0.04907249 |
| ULBP2 | 1.4209 | 0.01782911 | NME7 | -1.4491033 | 0.02802467 |
| CKMT1A | 1.37157333 | 0.01082086 | ZNF22 | -1.3659433 | 0.02909662 |
| C5orf51 | 1.32546667 | 0.0101758 | PCID2 | -1.343 | 0.03161522 |
| TANGO2 | 1.2655 | 0.01691057 | SOCS7 | -1.32889 | 0.00363703 |
| UNC13D | 1.25557667 | 0.01795457 | ARL14EP | -1.32881 | 0.02871909 |
| BLOC1S2 | BLOC1S2 | BLOC1S2 | TJAP1 | -1.31315 | 0.02491055 |
| VPS52 | VPS52 | VPS52 | RAD54L2 | -1.31109 | 0.04648084 |
| ZMYM1 | ZMYM1 | ZMYM1 | GIGYF1 | -1.30068 | 0.02554724 |
| ANAPC10 | ANAPC10 | ANAPC10 | PRDM4 | -1.2707667 | 0.03992842 |
| MORN2 | MORN2 | MORN2 | PREP | -1.2423667 | 0.0213679 |
| NGLY1 | NGLY1 | NGLY1 | SFXN4 | -1.23418 | 0.04046413 |
| ZMYM5 | ZMYM5 | ZMYM5 | TBC1D25 | -1.2004467 | 0.01509913 |
| SPC25 | SPC25 | SPC25 | PSIP1 | -1.19262 | 0.00455613 |
| STRADA | STRADA | STRADA | FIGNL1 | -1.13241 | 0.02510105 |
| TSNAX | TSNAX | TSNAX | NDE1 | -1.1077967 | 0.02709906 |
| NUDT1 | NUDT1 | NUDT1 | DUSP22 | -1.07745 | 0.01259594 |
| ZNF808 | ZNF808 | ZNF808 | PVT1 | -1.0521067 | 0.04040267 |
| GTPBP3 | GTPBP3 | GTPBP3 | IPO13 | -1.01806 | 0.01841356 |
| GRHL3 | GRHL3 | GRHL3 | CLP1 | -1.0166233 | 0.04480066 |
| ZNF880 | ZNF880 | ZNF880 | KBTBD4 | -1.00887 | 0.02199012 |
| C10orf82 | C10orf82 | C10orf82 | NUP133 | -0.9861333 | 0.03619557 |
| CLCA4 | CLCA4 | CLCA4 | PIK3CA | -0.9761333 | 0.03801437 |
| NCAPG | NCAPG | NCAPG | EN2 | -0.9547267 | 0.0051746 |
| MTM1 | MTM1 | MTM1 | TRIM39 | -0.94927 | 0.02286003 |
| C12orf4 | C12orf4 | C12orf4 | RBM18 | -0.93969 | 0.01269461 |
| VCPIP1 | VCPIP1 | VCPIP1 | GTF2IRD2 | -0.9293467 | 0.0312097 |
| SNHG15 | SNHG15 | SNHG15 | L3MBTL2 | -0.9218133 | 0.00199362 |
| NECTIN1 | NECTIN1 | NECTIN1 | ULK2 | -0.8970733 | 0.01606093 |
| PHACTR3 | 0.74239 | 0.02765792 | XRCC3 | -0.87817 | 0.01668748 |
| FASTKD1 | 0.70649333 | 0.00499504 | FAM89A | -0.8522933 | 0.03858827 |
| CAPN7 | 0.66266667 | 0.03004329 | IQCB1 | -0.83563 | 0.03889381 |
| FAM133B | 0.62640667 | 0.02952762 | ALKBH2 | -0.8266433 | 0.04713351 |
| CC2D1B | 0.61258333 | 0.04265106 | PXYLP1 | -0.8231333 | 0.01648486 |
| ZNF134 | 0.58195667 | 0.03297364 | ANGEL1 | -0.8226567 | 0.03913473 |
| ZNF350 | 0.55864667 | 0.01727289 | GPRIN1 | -0.8178867 | 0.04025463 |
| ENTPD8 | 0.49615233 | 0.0332783 | E2F1 | -0.8012633 | 0.04059799 |
| TRIM65 | 0.49452333 | 0.03715529 | TNFRSF1B | -0.79023 | 0.01380494 |
| IL17RE | 0.449866 | 0.04063543 | LZTFL1 | -0.7797667 | 0.03316682 |
| SNAI1 | 0.44421633 | 0.01478524 | IKZF3 | -0.7787683 | 0.00241131 |
| PUS10 | 0.40014 | 0.00971908 | YPEL2 | -0.7783133 | 0.04193844 |
| DGCR11 | 0.39787333 | 0.02140831 | ISY1 | -0.7614967 | 0.0322245 |
| PDE7A | 0.39509333 | 0.01879267 | CGRRF1 | -0.75344 | 0.03263131 |
| PLA2G7 | 0.380807 | 0.03752052 | ZNF518B | -0.7338333 | 0.03695317 |
| CAPN8 | 0.36257833 | 0.03332799 | TESMIN | -0.70501 | 0.00082119 |
| TSGA10 | 0.35592667 | 0.0452678 | C12orf65 | -0.69167 | 0.01718763 |
| ERC2 | 0.34484267 | 0.01094633 | MARS2 | -0.68802 | 0.00352164 |
| LY6G5B | 0.33878567 | 0.03726656 | DYNC1LI1 | -0.6713333 | 0.0348596 |
| WASH7P | 0.329942 | 0.0368127 | SNX10 | -0.6562433 | 0.04360616 |
| FAM86B3P | 0.32700033 | 0.02379167 | ACKR3 | -0.650631 | 0.02789282 |
| EFCAB7 | 0.31064333 | 0.03537519 | LGALS9DP | -0.6334667 | 0.01420284 |
| CDK20 | 0.30259 | 0.03910521 | SNRNP48 | -0.6221833 | 0.01914665 |
| ADH1C | 0.29988633 | 0.01196966 | DAPK2 | -0.6201433 | 0.02069111 |
| FUT6 | 0.292574 | 0.03069628 | POLE2 | -0.6178 | 0.04140472 |
| RCC2P6 | 0.28537233 | 0.00855188 | ZNF239 | -0.61562 | 0.00952399 |
| HACL1 | 0.27657 | 0.00493106 | TWNK | -0.61146 | 0.01679776 |
| TMEM88 | 0.23457967 | 0.02597591 | SEC61A2 | -0.6105867 | 0.03789638 |
| KLK12 | 0.20341767 | 0.02348299 | RARRES1 | -0.6018167 | 0.01692823 |
| PDZD7 | 0.19203733 | 0.00286771 | LIN37 | -0.5981867 | 0.00111441 |
| FOXF1 | 0.1900782 | 0.01936918 | LYSMD1 | -0.5850667 | 0.0455255 |
| DLG2 | 0.18844777 | 0.03629491 | RIN3 | -0.5783867 | 0.03541536 |
| MAFA | 0.18801527 | 0.00731726 | ABCB10 | -0.57405 | 0.02548709 |
| AZIN2 | 0.18391933 | 0.0044904 | FXN | -0.5729367 | 0.04558001 |
| GJB4 | 0.177449 | 0.0218061 | MTHFSD | -0.5663 | 0.01823347 |
| RPSAP70 | 0.1644498 | 0.00646967 | MVB12B | -0.5640447 | 0.0371545 |
| MED14OS | 0.1600981 | 0.01712962 | ELMOD3 | -0.5635733 | 0.00498705 |
| ANKUB1 | 0.15977067 | 0.00780957 | KRI1 | -0.5583133 | 0.00878061 |
| NR4A3 | 0.15825867 | 0.02606097 | CENPP | -0.5510497 | 0.0173271 |
| SCN8A | 0.15257773 | 0.04938262 | PCGF6 | -0.5369267 | 0.03533618 |
| NME9 | 0.15237033 | 0.0305623 | GID4 | -0.5315733 | 0.03401083 |
| FAM95C | 0.14914727 | 0.00168234 | TUBD1 | -0.5257633 | 0.03561757 |
| C4B | 0.14345533 | 0.04857511 | ZNF717 | -0.5127933 | 0.02745281 |
| MGAT5B | 0.13865033 | 0.03210691 | ZNF490 | -0.50571 | 0.04446784 |
| CCRL2 | 0.127701 | 0.03521197 | ZNF16 | -0.50451 | 0.00916191 |
| DRC3 | 0.12581167 | 0.03632849 | ABCB9 | -0.499552 | 0.00458146 |
| RAB7B | 0.12573467 | 0.02918652 | ANKRD44 | -0.4964963 | 0.01985511 |
| NPM1P37 | 0.12524503 | 0.00509081 | KLHL23 | -0.47827 | 0.03440104 |
| C6 | 0.12292973 | 0.00194516 | ZNF778 | -0.4780367 | 0.04178304 |
| TCEA1P4 | 0.12275267 | 0.04394682 | FAM109B | -0.4757233 | 0.01431272 |
| CBLN3 | 0.11473667 | 0.03499206 | PDE4A | -0.45708 | 0.00808882 |
| LY6D | 0.11462897 | 0.01321083 | NPHP4 | -0.4513633 | 0.00212843 |
| WFDC12 | 0.11314067 | 0.02789684 | MOCS1 | -0.4417667 | 0.00781318 |
| PTPN22 | 0.11281533 | 0.03836837 | RPL29P11 | -0.4284253 | 0.00915493 |
| IDSP1 | 0.1108776 | 0.01374593 | RPS6KL1 | -0.4267493 | 0.00797619 |
| PDLIM1P4 | 0.10685263 | 0.01962467 | TTI1 | -0.4172833 | 0.02393377 |
| VN1R1 | 0.1028065 | 0.00333945 | CD83 | -0.4142023 | 0.01958635 |
| NOXRED1 | 0.1023291 | 0.03542032 | SLC46A3 | -0.4089767 | 0.00528496 |
| DUSP8P3 | 0.10189717 | 0.0037739 | TRIM45 | -0.4056457 | 0.00233962 |
| RAB1C | 0.10185767 | 0.0030974 | JPH1 | -0.400575 | 0.01087689 |
|  |  |  | CBX2 | -0.3873433 | 0.00867667 |
|  |  |  | LRRC27 | -0.3853733 | 0.0120302 |
|  |  |  | PASK | -0.3742433 | 0.02622824 |
|  |  |  | FRAT1 | -0.3533723 | 0.02283602 |
|  |  |  | TMEM81 | -0.3533313 | 0.01511711 |
|  |  |  | SLC27A2 | -0.352081 | 0.01442258 |
|  |  |  | MAML3 | -0.35189 | 0.04509578 |
|  |  |  | TEX101 | -0.3460088 | 0.01100707 |
|  |  |  | ZXDA | -0.3423647 | 0.02286194 |
|  |  |  | MMACHC | -0.3419167 | 0.01485764 |
|  |  |  | SLC28A3 | -0.332961 | 0.02387811 |
|  |  |  | CKS1BP3 | -0.3254853 | 0.04650054 |
|  |  |  | HYKK | -0.3204467 | 0.01670966 |
|  |  |  | TOP1MT | -0.31239 | 0.03684593 |
|  |  |  | CDK5R1 | -0.3036453 | 0.01624084 |
|  |  |  | CDAN1 | -0.3028 | 0.00950068 |
|  |  |  | AADAT | -0.30153 | 0.04394115 |
|  |  |  | MISP3 | -0.295097 | 0.04858313 |
|  |  |  | BMP6 | -0.294078 | 0.00521885 |
|  |  |  | MYBL1 | -0.28599 | 0.01425661 |
|  |  |  | SHPRH | -0.2834933 | 0.0213235 |
|  |  |  | ZNF747 | -0.2762 | 0.00804023 |
|  |  |  | SPIN4 | -0.26609 | 0.02991422 |
|  |  |  | RUNX3 | -0.265409 | 0.02385697 |
|  |  |  | FAM86C2P | -0.2637297 | 0.04983281 |
|  |  |  | ZNF304 | -0.2624367 | 0.02519193 |
|  |  |  | CDKL3 | -0.2408797 | 0.04617897 |
|  |  |  | DHX40P1 | -0.2262203 | 0.01331067 |
|  |  |  | EME1 | -0.2261633 | 0.04925909 |
|  |  |  | ZNF180 | -0.2204667 | 0.03687696 |
|  |  |  | WNT3 | -0.2182683 | 0.03509997 |
|  |  |  | SLC29A3 | -0.21547 | 0.01265681 |
|  |  |  | ZNF829 | -0.2134537 | 0.00552707 |
|  |  |  | TTC21A | -0.2126807 | 0.04380642 |
|  |  |  | PHC1P1 | -0.2015903 | 0.001472 |
|  |  |  | CDC42P6 | -0.1967463 | 0.02005808 |
|  |  |  | ETFBKMT | -0.1885053 | 0.0307127 |
|  |  |  | RGS6 | -0.18461 | 0.04220613 |
|  |  |  | HSPBAP1 | -0.1825967 | 0.04363759 |
|  |  |  | ZNF483 | -0.1780893 | 0.00332113 |
|  |  |  | BTF3L4P2 | -0.1770467 | 0.00298052 |
|  |  |  | TP73 | -0.159919 | 0.0138852 |
|  |  |  | ZKSCAN7 | -0.157434 | 0.01938327 |
|  |  |  | TAS2R43 | -0.1563437 | 0.01118716 |
|  |  |  | RNF7P1 | -0.1555264 | 0.02877047 |
|  |  |  | HLA-DQA1 | -0.146135 | 0.04233274 |
|  |  |  | KCNJ14 | -0.1457837 | 0.02540278 |
|  |  |  | CFAP126 | -0.1370723 | 0.04700504 |
|  |  |  | SYNGR3 | -0.135567 | 0.03404052 |
|  |  |  | GLOD5 | -0.1339042 | 0.03880977 |
|  |  |  | GPR141 | -0.1306519 | 0.00416162 |
|  |  |  | ANKRD35 | -0.1234243 | 0.03063356 |
|  |  |  | CLUHP3 | -0.1206567 | 0.03356209 |
|  |  |  | ALS2CR12 | -0.1203935 | 0.00464591 |
|  |  |  | PANO1 | -0.1108186 | 0.03552093 |
|  |  |  | NACAD | -0.1081787 | 0.01555702 |

| **Table S4 Gene Ontology (GO) enrichment analyses** | | | |
| --- | --- | --- | --- |
| **Upregulated genes** | | **Downregulated genes** | |
| **Biological processes** | **P Value** | **Biological processes** | **P Value** |
| Positive regulation of immune effector process | 0.0012 | Cellualr aromatic compound metabolic process | 0.0049 |
| Mast cell activation involved in immune response | 0.0047 | Heterocycle metabolic process | 0.0062 |
| Regulation of immune effector process | 0.0092 | Chromosome segregation | 0.0064 |
| Negative regulation of autophagy | 0.011 | Organic cyclic compound metabolic process | 0.0074 |
| Leukocyte degranulation | 0.012 | Nucleobase-containing compound metabolic process | 0.0085 |
| Leukocyte mediated immunity | 0.015 | Negative regulation of developmental growth | 0.012 |
| Myeloid leukocyte immunity | 0.022 | Regulation of cell death | 0.015 |
|  |  | Cellular nitrogen compound metabolic process | 0.015 |
|  |  | Regulation of autophagy | 0.018 |
|  |  | Regulation of leukocyte activation | 0.019 |
|  |  | RNA localization | 0.019 |
|  |  | Nuclear chromosome segregation | 0.025 |
|  |  | Regulation of cell activation | 0.029 |
|  |  | Glycosyl compound metabolic process | 0.030 |
|  |  | Cellular biosynthetic process | 0.032 |
|  |  | Regulation of developmental growth | 0.032 |
|  |  | Estabalishment of RNA localization | 0.034 |
|  |  | Chromosome separation | 0.036 |
|  |  | Ribonucleoprotein complex localization | 0.036 |
|  |  | Regulation of nitrogen compound metabolic process | 0.046 |
