# Supplementary figures and images for: ΔNp63α-induced DUSP4/GSK3β/SNAI1 pathway in epithelial cells drives endometrial fibrosis
Source: Cell Death Dis. 2020 Jun 11;11(6):449. doi: 10.1038/s41419-020-2666-y (PMC7289806; doi:10.1038/s41419-020-2666-y)

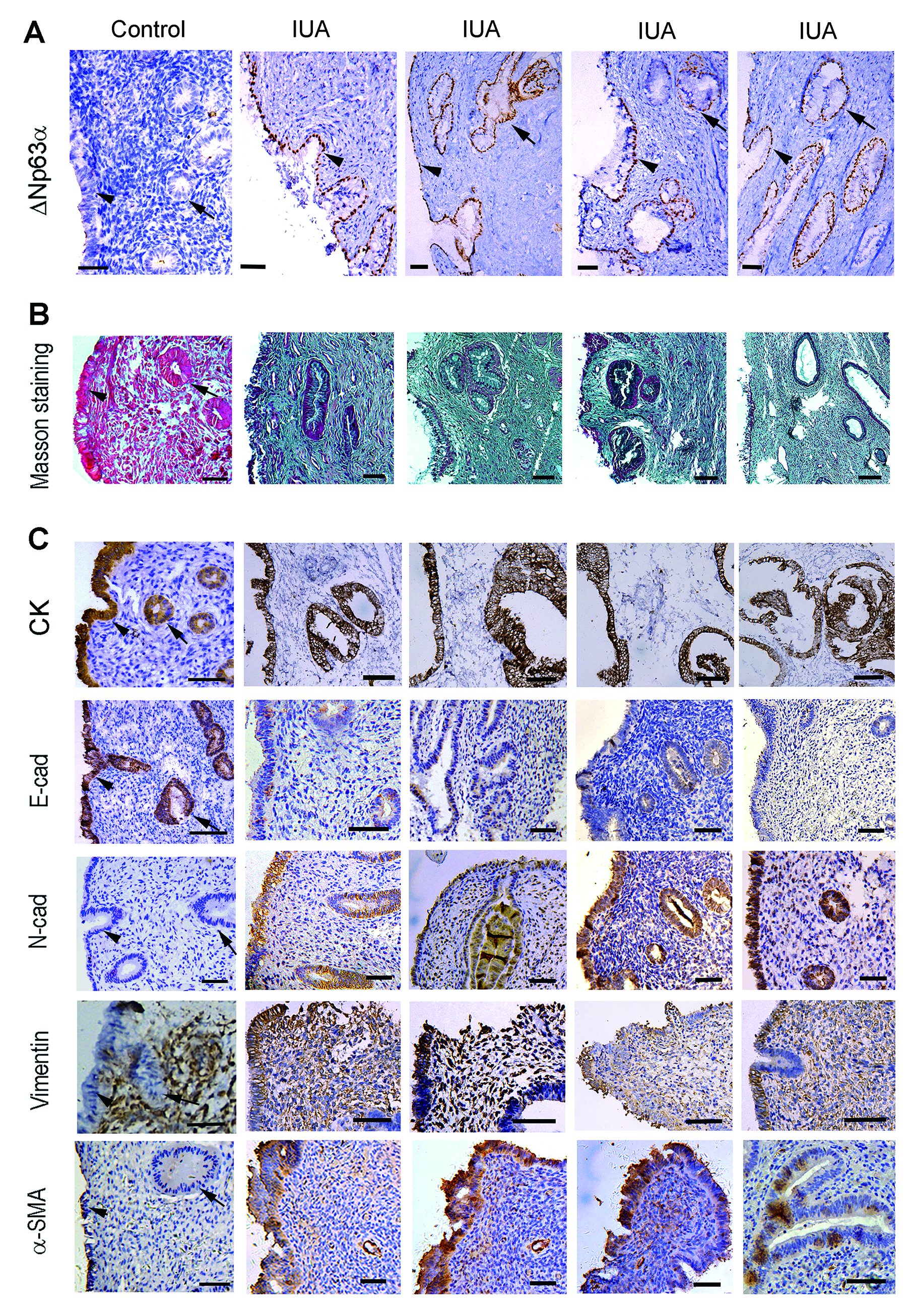

Supplement: Supplementary file 3 — Supp. Figure 1 [file 41419_2020_2666_MOESM3_ESM.tif]

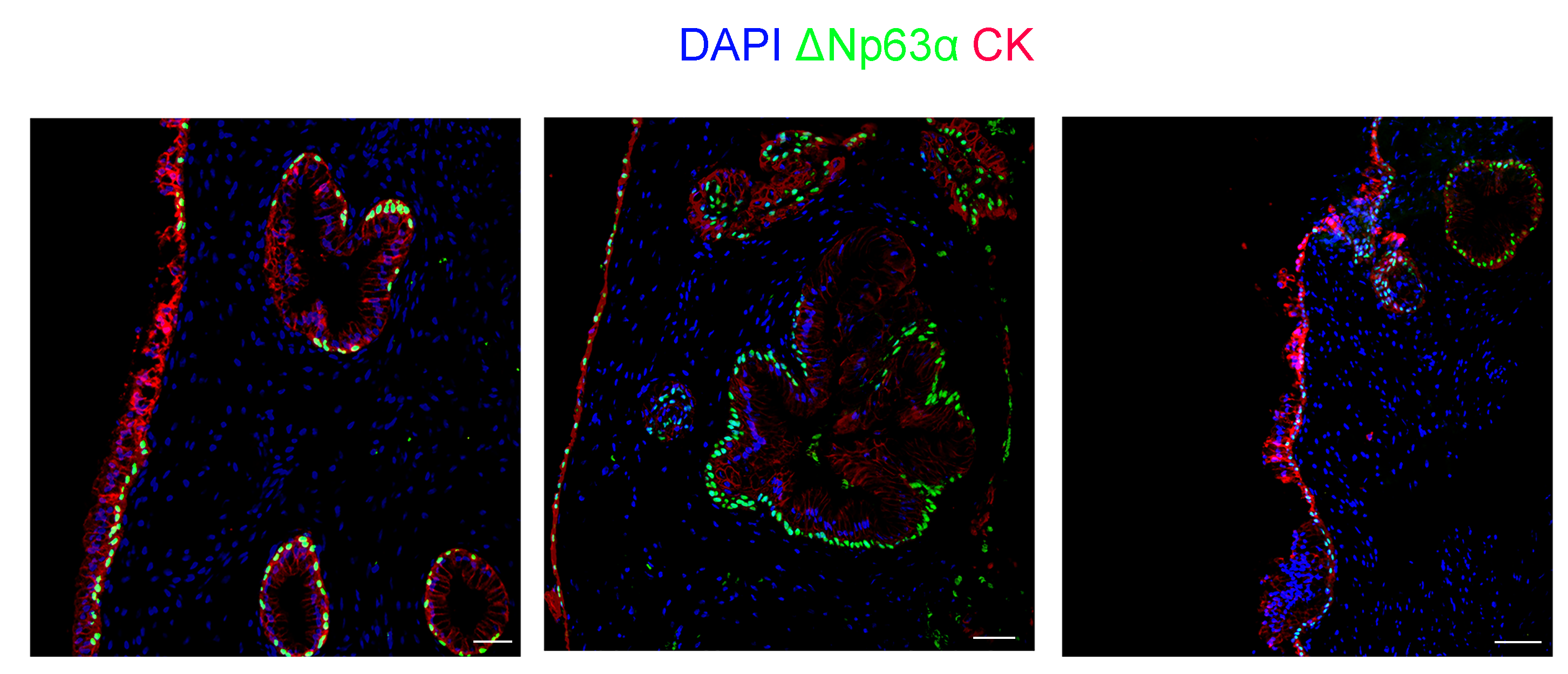

Supplement: Supplementary file 4 — Supp. Figure 2 [file 41419_2020_2666_MOESM4_ESM.tif]

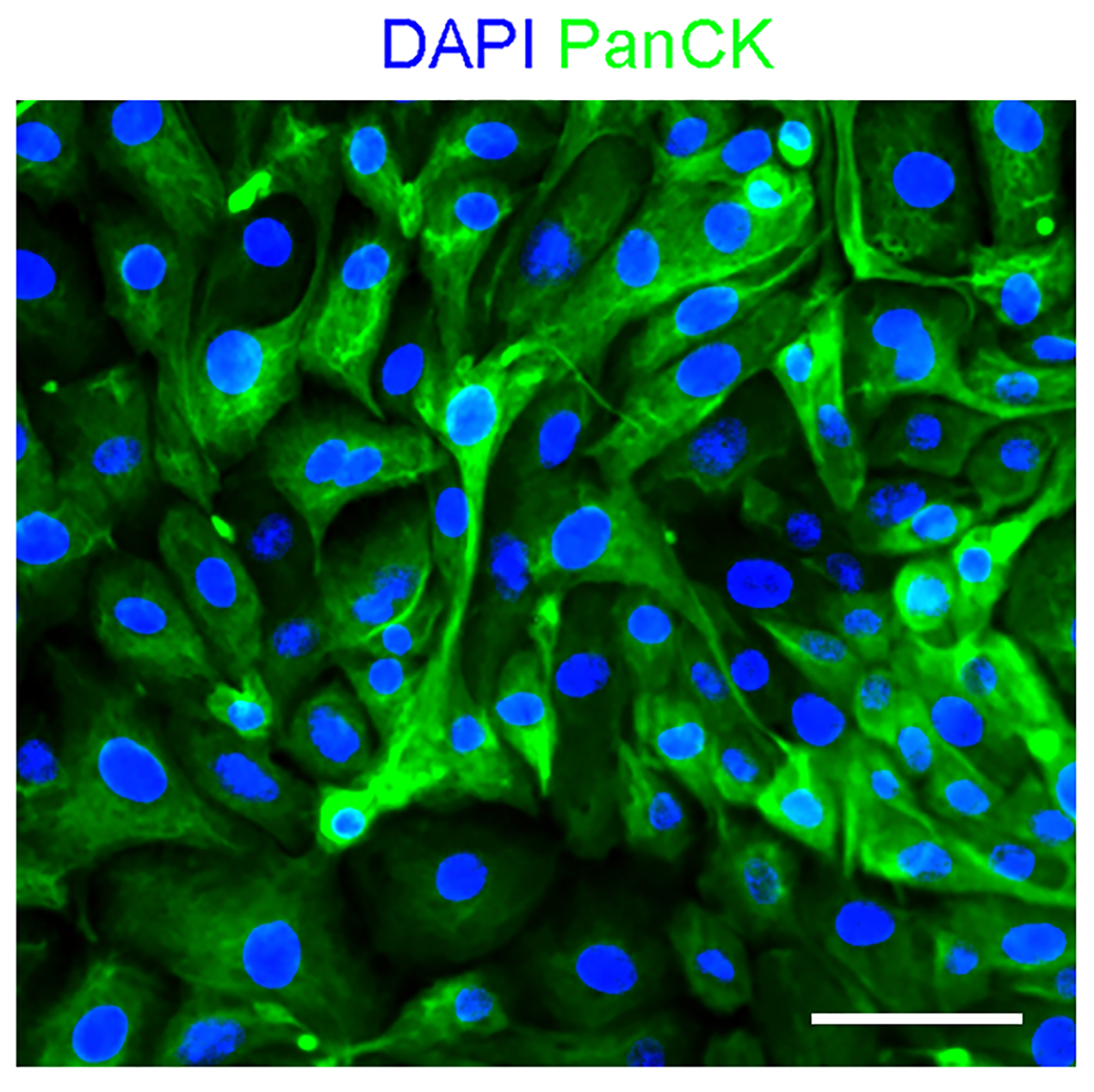

Supplement: Supplementary file 5 — Supp. Figure 3 [file 41419_2020_2666_MOESM5_ESM.tif]

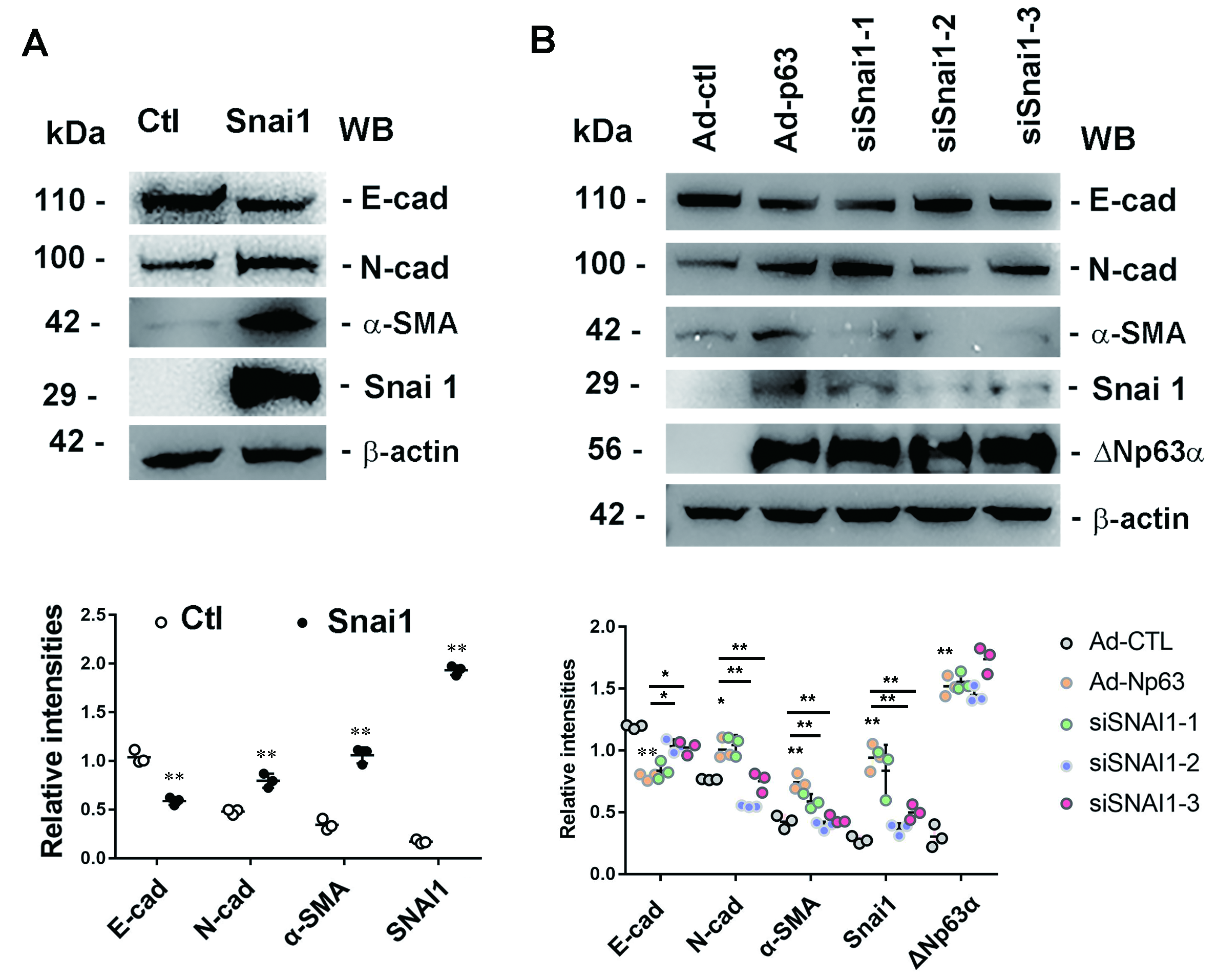

Supplement: Supplementary file 6 — Supp. Figure 5 [file 41419_2020_2666_MOESM6_ESM.tif]

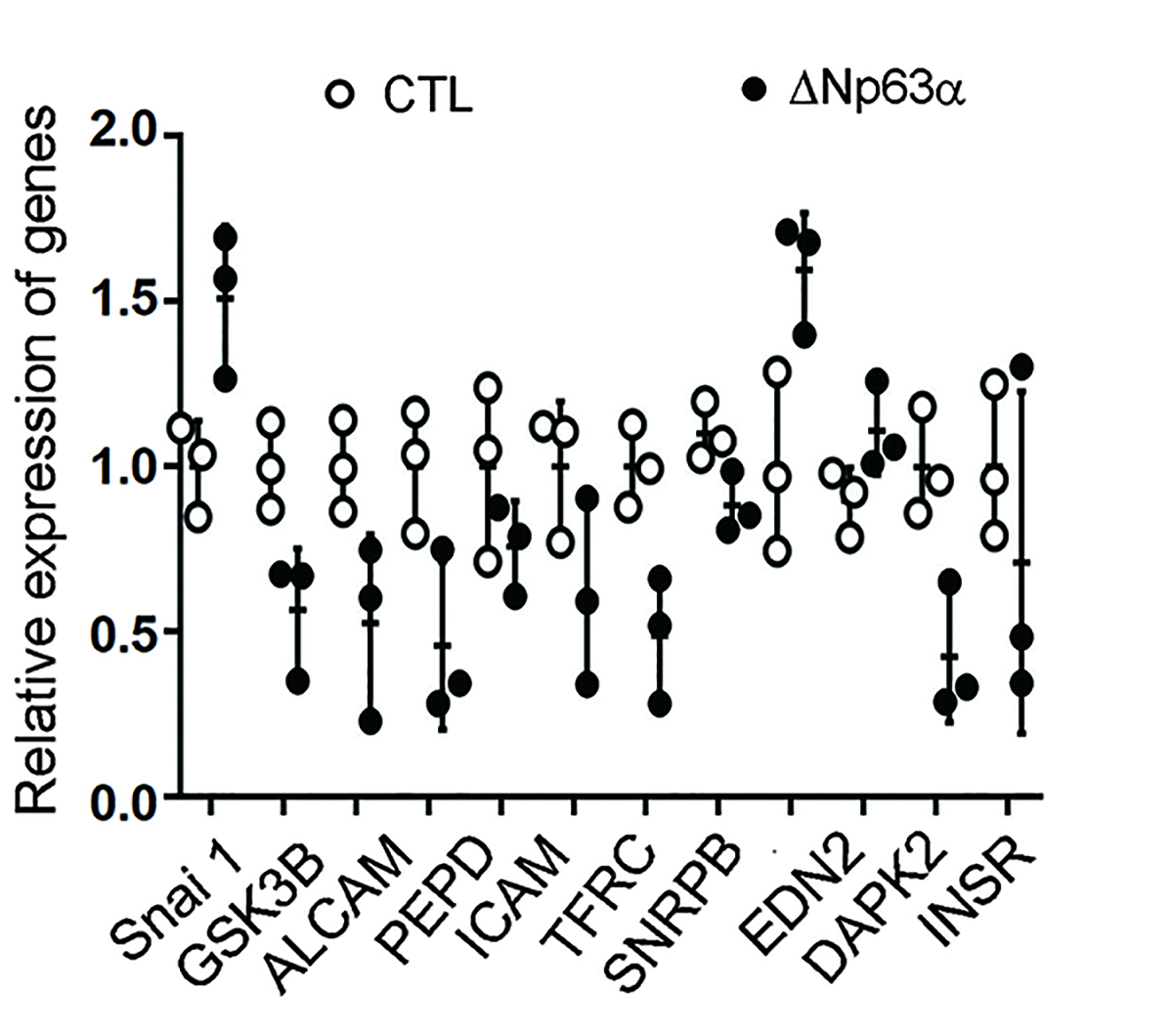

Supplement: Supplementary file 7 — Supp. Figure 4 [file 41419_2020_2666_MOESM7_ESM.tif]

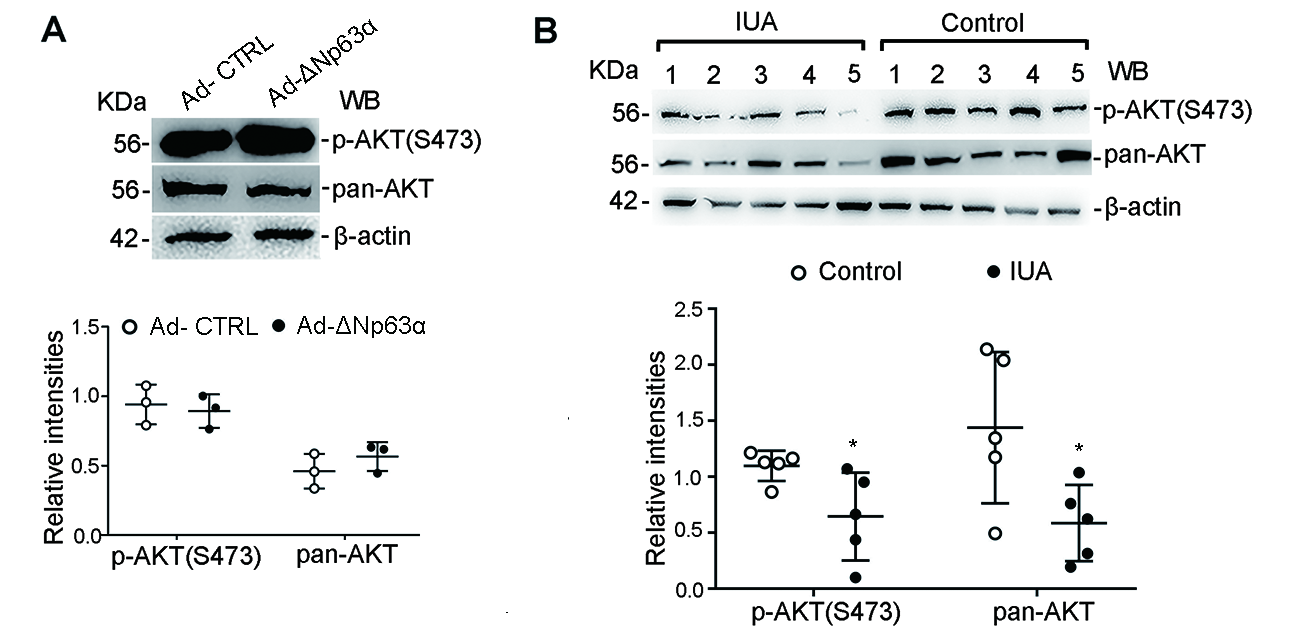

Supplement: Supplementary file 8 — Supp. Figure 6 [file 41419_2020_2666_MOESM8_ESM.tif]

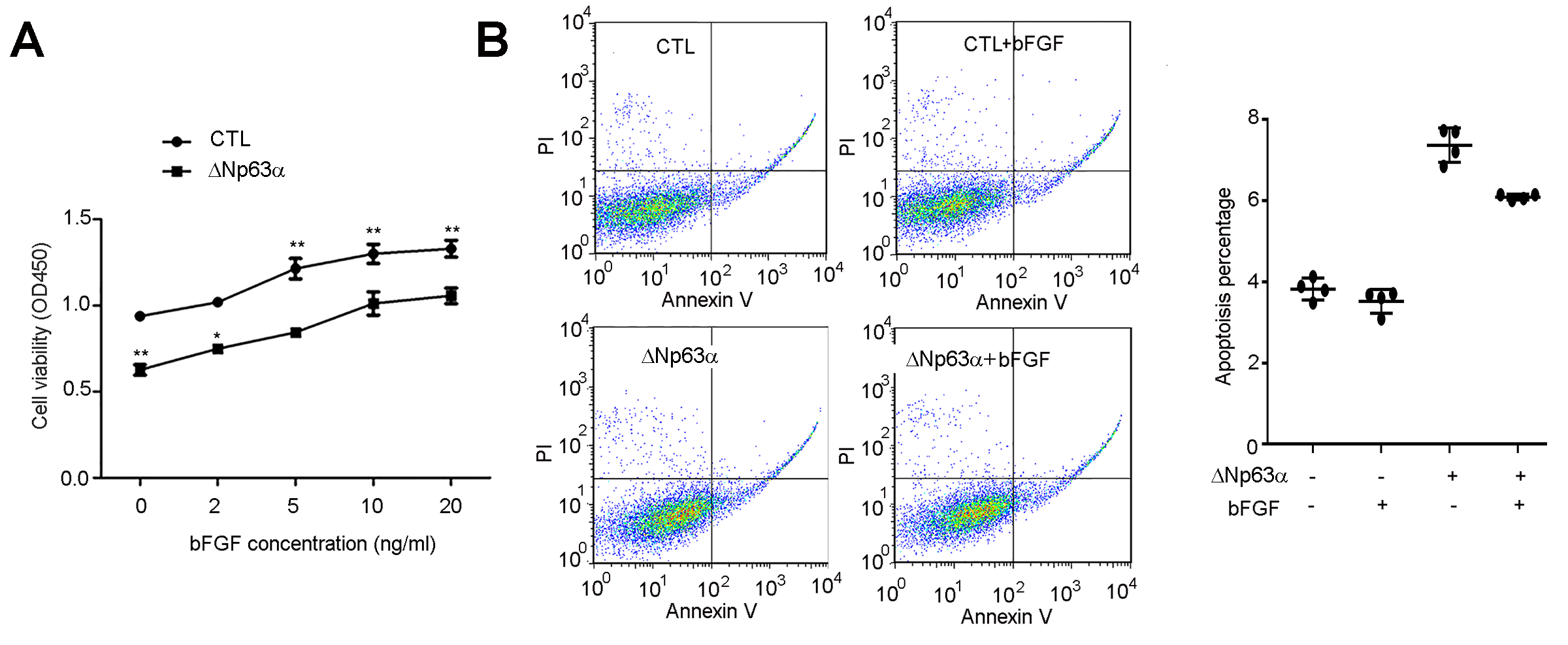

Supplement: Supplementary file 9 — Supp. Figure 7 [file 41419_2020_2666_MOESM9_ESM.tif]

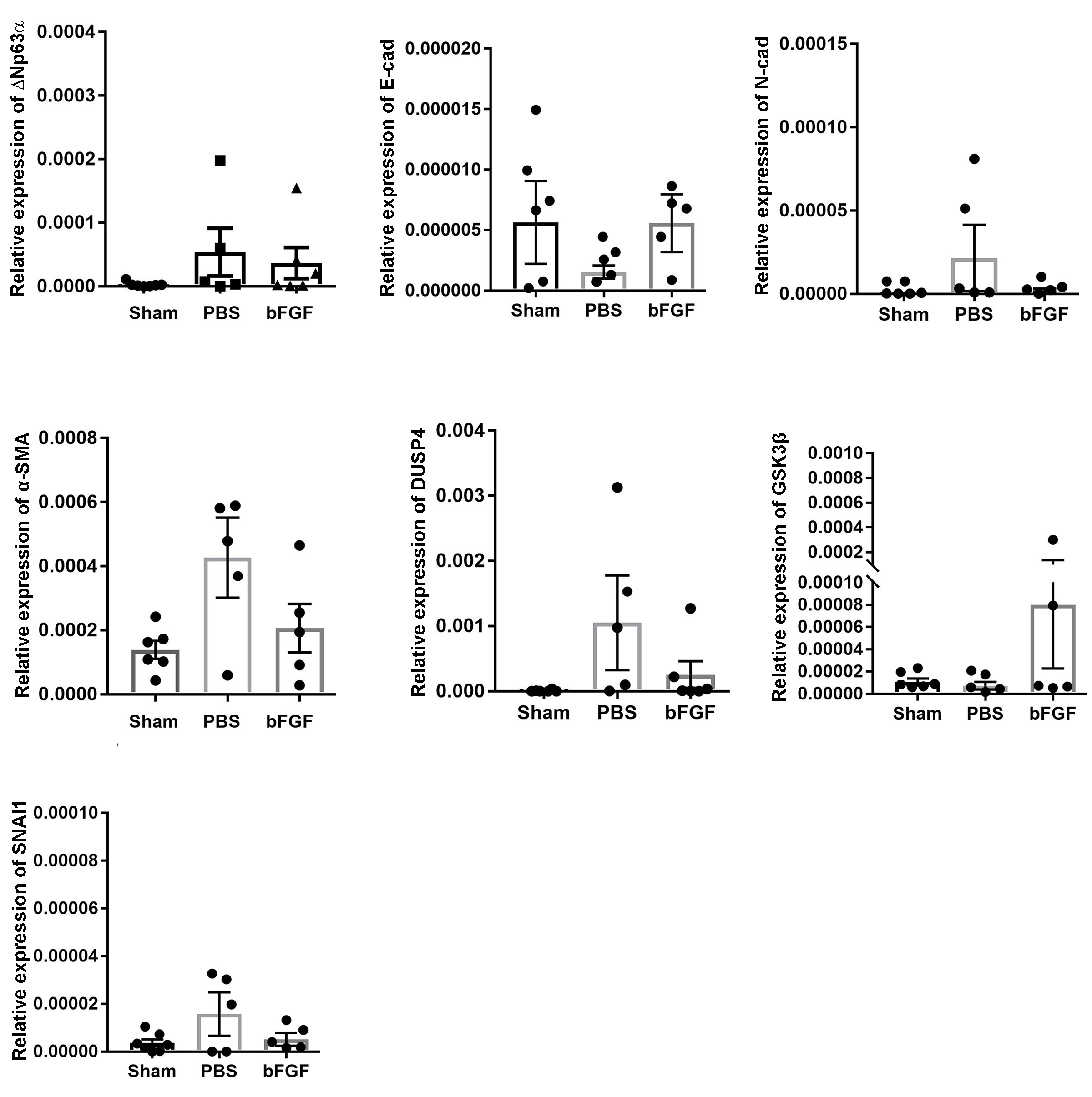

Supplement: Supplementary file 10 — Supp. Figure 8 [file 41419_2020_2666_MOESM10_ESM.tif]
